# Marker-Free Tracking for Motion Artifact Compensation and Deformation Measurements in Optical Mapping Videos of Contracting Hearts

**DOI:** 10.3389/fphys.2018.01483

**Published:** 2018-11-02

**Authors:** Jan Christoph, Stefan Luther

**Affiliations:** ^1^Biomedical Physics Group, Max Planck Institute for Dynamics and Self-Organization, Göttingen, Germany; ^2^German Center for Cardiovascular Research, Göttingen, Germany; ^3^Institute for Nonlinear Dynamics, University of Göttingen, Göttingen, Germany; ^4^Department of Pharmacology, University Medical Center, University of Göttingen, Göttingen, Germany

**Keywords:** fluorescence imaging, optical mapping, motion tracking, computer vision, cardiac arrhythmias, ventricular fibrillation, atrial fibrillation, heart rhythm disorders

## Abstract

Optical mapping is a high-resolution fluorescence imaging technique, which provides highly detailed visualizations of the electrophysiological wave phenomena, which trigger the beating of the heart. Recent advancements in optical mapping have demonstrated that the technique can now be performed with moving and contracting hearts and that motion and motion artifacts, once a major limitation, can now be overcome by numerically tracking and stabilizing the heart's motion. As a result, the optical measurement of electrical activity can be obtained from the moving heart surface in a co-moving frame of reference and motion artifacts can be reduced substantially. The aim of this study is to assess and validate the performance of a 2D marker-free motion tracking algorithm, which tracks motion and non-rigid deformations in video images. Because the tracking algorithm does not require markers to be attached to the tissue, it is necessary to verify that it accurately tracks the displacements of the cardiac tissue surface, which not only contracts and deforms, but also fluoresces and exhibits spatio-temporal physiology-related intensity changes. We used computer simulations to generate synthetic optical mapping videos, which show the contracting and fluorescing ventricular heart surface. The synthetic data reproduces experimental data as closely as possible and shows electrical waves propagating across the deforming tissue surface, as seen during voltage-sensitive imaging. We then tested the motion tracking and motion-stabilization algorithm on the synthetic as well as on experimental data. The motion tracking and motion-stabilization algorithm decreases motion artifacts approximately by 80% and achieves sub-pixel precision when tracking motion of 1–10 pixels (in a video image with 100 by 100 pixels), effectively inhibiting motion such that little residual motion remains after tracking and motion-stabilization. To demonstrate the performance of the algorithm, we present optical maps with a substantial reduction in motion artifacts showing action potential waves propagating across the moving and strongly deforming ventricular heart surface. The tracking algorithm reliably tracks motion if the tissue surface is illuminated homogeneously and shows sufficient contrast or texture which can be tracked or if the contrast is artificially or numerically enhanced. In this study, we also show how a reduction in dissociation-related motion artifacts can be quantified and linked to tracking precision. Our results can be used to advance optical mapping techniques, enabling them to image contracting hearts, with the ultimate goal of studying the mutual coupling of electrical and mechanical phenomena in healthy and diseased hearts.

## 1. Introduction

Optical mapping is a high-resolution fluorescence imaging technique, which is widely used in basic cardiovascular science (Herron et al., 2012). It employs optical probes or fluorophores, excitation light, high-speed cameras and filtering equipment and is typically used to image the electrophysiological activity that triggers the beating of the heart. With voltage- and calcium-sensitive dyes, for instance, it is possible to image action potential and calcium waves propagating across the heart surface in great detail and at very high speeds. To avoid undesired motion artifacts during such highly sensitive measurements, it has been necessary to suppress the beating of the heart during optical mapping by using pharmacological excitation-contraction uncoupling substances such as Blebbistatin (Fedorov et al., 2007) or DAM. Recent developments, however, have demonstrated that action potential and calcium waves can also be imaged as they propagate across the strongly contracting and deforming heart surface (Zhang et al., 2016; Christoph et al., 2017, 2018). In combining optical mapping with computer vision techniques and numerically tracking the heart's motion, the optical imaging of electrical activity during heart contraction is possible. The tracking inherently also allows the measurement of the cardiac deformation and thus the mechanical activity. The simultaneous imaging of both the heart's contractile motion and the electrochemical processes that generate the heart's contractions is pivotal for a better understanding of the heart's electrophysiology and mechanics and their mutual coupling.

In this work, we validate and discuss the performance of a 2D numerical motion tracking and motion compensation algorithm, which reliably tracks both the heart's rigid and non-rigid body motion and planar movements within video images obtained with a single camera during optical mapping. In previous studies, we used the algorithm to map action potential waves during sinus rhythm on the contracting three-dimensional heart surface using multiple cameras (Christoph et al., 2017) and to map arrhythmic action potential and calcium vortex waves during ventricular tachycardia and fibrillation on the surface of contracting rabbit and pig hearts (Christoph et al., 2018). Processing various optical mapping recordings obtained with different species and sensitivities (Di-4-ANEPPS, Di-4-ANBDQPQ, Rhod-2AM), we were able to retrieve motion-stabilized optical maps and co-moving optical traces, in which the fluorescent signals could be measured along a trajectory describing the movement of the tissue through the video image. After motion-stabilization, we were able to measure sequences of action potentials and calcium transients and their spatio-temporal evolution across the moving heart surface with a substantial reduction in dissociation-related motion artifacts. Dissociation-related motion artifacts occur due to a loss of the correspondence between a particular pixel of the camera sensor and a particular piece of cardiac tissue that is imaged with the pixel when the tissue moves. Furthermore, using the tracking data, we were able to measure and analyze the rapid mechanical deformations that the ventricular cardiac muscle exhibits during fibrillation, and were able to relate elasto-mechanical patterns arising in the heart wall to the turbulent electrical activity that causes the heart's fibrillatory contractions (Christoph et al., 2018). While the aim of our previous multi-camera study (Christoph et al., 2017) was to provide a proof-of-concept that three-dimensional electromechanical optical mapping is possible, our aim in the present study is to discuss the performance of the 2D tracking itself. In this study, we carefully assess and demonstrate the algorithm's efficacy and robustness in reliably detecting shifts of the tissue in the video images using both experimental and synthetic optical mapping data generated with computer simulations. In particular, the synthetic optical mapping data allows the comparison of the tracking outcomes to ground-truth data, as it becomes possible to precisely measure mismatches between the simulated and tracked tissue configurations. We reproduced experimental data as closely as possible and used the electromechanical computer simulations to mimic key video properties such as different contraction strengths, image contrasts and fractional intensity changes of the fluorescence. We then used the simulations to systematically generate optical mapping videos containing motion and motion artifacts and applied the tracking algorithm to track and stabilize the motion and remove the motion artifacts under various conditions.

## 2. Materials and methods

Experimental and synthetic optical mapping video data was generated and analyzed, the video data showing the contracting and fluorescing heart surface filmed through a monocular imaging setup with one camera. In particular, video data with varying amplitudes of motion and fluorescent signal strengths was analyzed.

### 2.1. Experimental setup and imaging protocol

**Figure 1:**
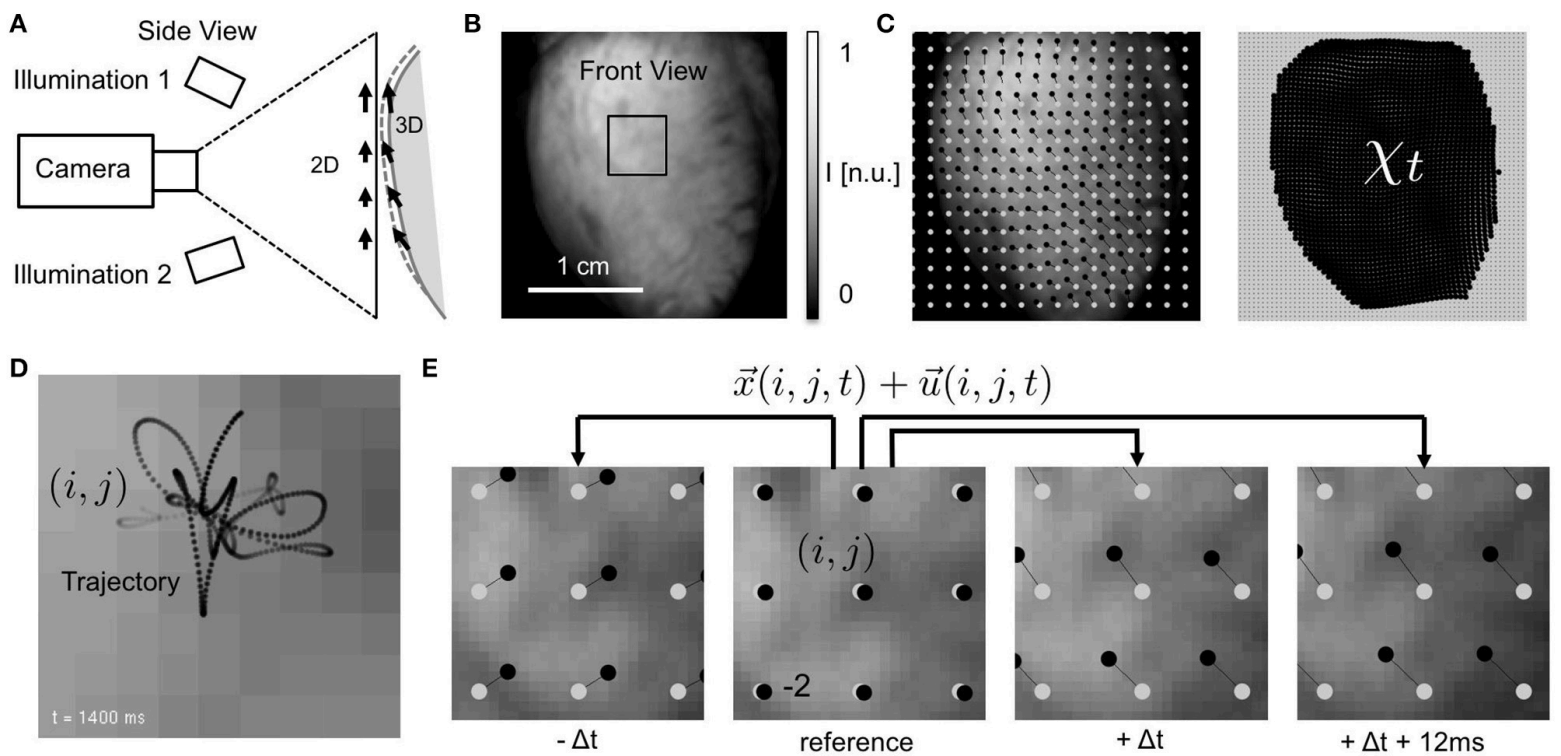
Electromechanical optical mapping with beating, strongly deforming and fluorescing hearts. **(A)** Schematic drawing of experimental setup with single high-speed camera filming ventricular surface of rabbit heart (side view). Tracking of motion is possible only within the image plane (2D). **(B)** Raw video image (128 × 128 pixels) showing heart surface during optical mapping with voltage-sensitive staining (front view). ROI see **(E)**. **(C)** Tracked displacements and mechanical configuration χ_*t*_ at time *t* (left: every 8*th* vector, right: every 2*nd* vector). Points (black) indicating displacements of single tissue segment with respect to reference position (gray). See also Supplementary Video 1. **(D)** Trajectory of tracked tissue segment moving during ventricular fibrillation through pixel plane. Motion amplitudes are in the order of a few pixels (maximal 3–4 pixels from initial position, 2 ms temporal resolution). **(E)** Tracking of tissue movements during pacing with displacement amplitudes in the order of $|\vec{u}|\approx 10.0$ pixels. Points (black) indicating movements of single tissue segment (*i, j*) with respect to reference position (gray) in reference video image *I*_*r*_ (shown is every 10*th* segment or vector for illustration purposes).

Contracting isolated Langendorff-perfused rabbit hearts (*N* = 2) were filmed during regular rhythm and ventricular arrhythmias using a single-camera optical mapping system, see Figure 1. Pharmacological excitation-contraction uncoupling agents such as Blebbistatin were intentionally not administered. Any other mechanical constraints were avoided to let the heart beat freely. The optical mapping system consisted of a single EMCCD camera (Evolve 128, Photometrics Inc., 128 × 128 pixels, 16 bit dynamic range), using a high-aperture lens (objective Fujinon 1.4/9*mm*, approx. 2 × 2*cm* field of view, Fujifilm Corp.) and long-pass filtering (Edmund Optics, transmission > 610*nm*) to filter the fluorescent light emitted from the heart surface. Hearts were stained with voltage-sensitive dye (Di-4-ANEPPS, 20*ml* of 1*mMol*/*l* concentrated dye-Tyrode solution, 605*nm* emission peak, bolus injection, recirculated). The dye was excited using four light-emitting diodes operating at wavelengths of 532*nm*, powered by batteries (12*V*, 26*Ah*, rechargeable) to maintain constant, low-noise illumination. The diodes were positioned close to and around the camera lens and directed onto the central part of the ventricular wall, to establish a homogeneous illumination. For even distribution of the dye in the tissue, filming was started not earlier than 5 min after the dye was administered. Camera triggers were provided from an external triggering source (wave form generator, 33220A, Agilent) and recordings were obtained at a frame rate of *fps* = 500*Hz*. Hearts were excised from anesthetized New Zealand white rabbits (*N* = 2, female, 6–12 months, 2.5 − 3.5*kg*) and inserted into cardioplegic solution for temporary cessation of cardiac activity. This study was carried out in accordance with German animal welfare laws and the recommendations of the Lower Saxony State Office for Customer Protection and Food Safety (LAVES) and the Federation of European Laboratory Animal Science Associations (FELASA). The protocol was approved by the Lower Saxony State Office for Customer Protection and Food Safety (LAVES). The hearts were positioned at the center of a 8-sided, glass-walled bath filled with oxygenated 37° warm Tyrode solution (95% O 2, 5% CO 2) and connected to a retrograde Langendorff-perfusion system (Hugo-Sachs Apparatus, March-Hugstetten, Germany). The flow rate of the perfusate was 30*mlmin*^−1^ at a perfusion pressure of 50*mmHg* ± 5*mmHg*. The Tyrode solution was kept at a constant temperature of 37°*C* ± 0.5°*C* (custom-made temperature control, Max Planck Institute for Dynamics and Self-Organization, Göttingen, Germany) and was constantly reperfused. Hearts were attached at the aorta to the retrograde perfusion outflow, hanging vertically from the aortic block, the apex facing the bottom of the bath. The camera was positioned at heart level and filmed the epicardial ventricular surface through one of the glass walls of the bath. Filming was performed at working distances of approximately *d* = 30*cm*. Hearts were filmed with their ventricular surface facing the camera, see Figure 1A. Mechanical pressure on the hearts was carefully avoided to prevent compression of the coronary arteries. Electrocardiograms were recorded using a data acquisition system (MP150, Biopac Systems Inc., Goleta, USA), acquiring data at a sampling rate of 2.0*kHz* throughout the entire duration of the experiment.

### 2.2. Synthetic optical mapping videos generated with computer simulations

To be able to assess the efficacy of motion tracking and motion artifact compensation algorithms systematically, synthetic optical mapping video data was generated using computer simulations. A modified two-dimensional numerical reaction-diffusion mechanics model (Weise et al., 2011; Christoph, 2015) was used to create maps of electrical action potential wave patterns on a correspondingly contracting and deforming two-dimensional elastic surface, see section 2.2.1. The simulation data was used to deform a video image showing the heart surface during an optical mapping experiment with voltage-sensitive staining (Di-4-ANEPPS), and to modulate its pixel intensities according to the model's transmembrane voltage or electrical wave pattern, see section 2.2.2. Tracking and motion compensation was then applied to the synthetically generated optical mapping video data.

#### 2.2.1. Numerical model

**Figure 2:**
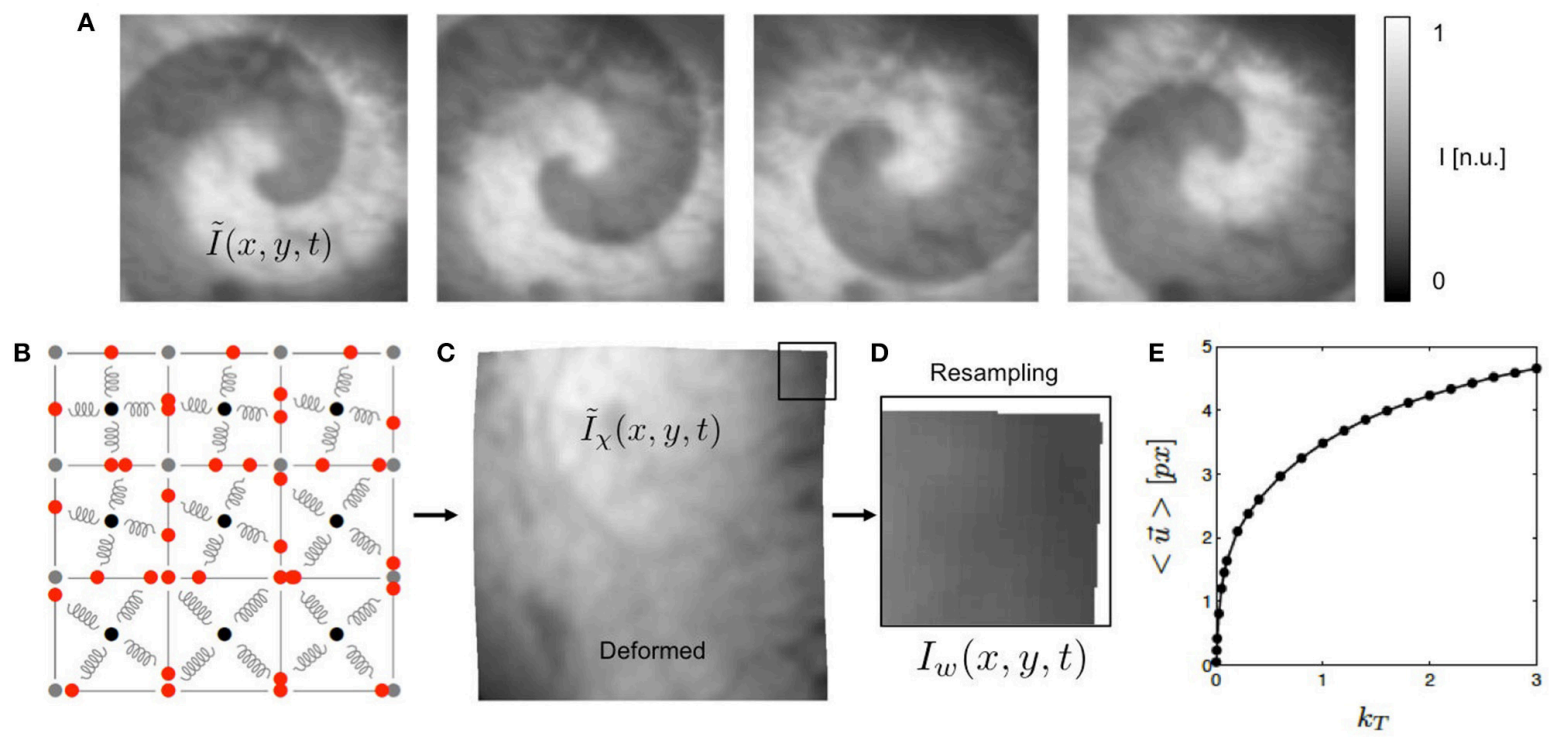
Generation of synthetic optical maps using computer simulations. **(A)** Original optical mapping image (100 × 100 pixels) cropped from video still frame showing heart surface of rabbit heart superimposed by simulated spiral wave pattern (exaggerated) generated using reaction-diffusion excitable dynamics model. Simulated fluorescence intensity drop during depolarization of action potential (exaggerated). **(B)** Deformable elastic medium simulated with mass-spring damper lattice model (here consisting of only 3 × 3 cells for illustration purposes, system used in study consists of 100 × 100 cells). Each pixel in **(A)** corresponds to one cell. **(C)** Deformed video image Ĩ_χ_(*x, y*) containing fluorescent signal (Δ*F*/*F* < 5%, not visible in a still frame), deformation caused by spiral dynamics. See also Supplementary Video 3. **(D)** Deformed and resampled part of video image *I*(*x, y*). **(E)** Overall displacements of simulation in pixels depending on simulation parameter *k*_*T*_ to modulate contraction strength of springs.

A two-dimensional elastic excitable medium with tunable muscle fiber anisotropy was used to produce nonlinear waves of excitation propagating in a correspondingly deforming two-dimensional elastic medium, see Figures 2A,B. The elastic excitable medium consists of two numerical models, an electrical and an elastic model, coupled using forward electromechanical coupling. The electrical model allows the simulation of electrical impulse propagation, such as planar or target wave patterns, and also produces spiral wave patterns or chaotic wave activity composed of multiple spiral waves, as similarly observed in optical mapping experiments on the heart surface during arrhythmias. Due to the forward electromechanical coupling the electrical wave patterns cause local contractions and deformations of the elastic medium.

**Figure 3:**
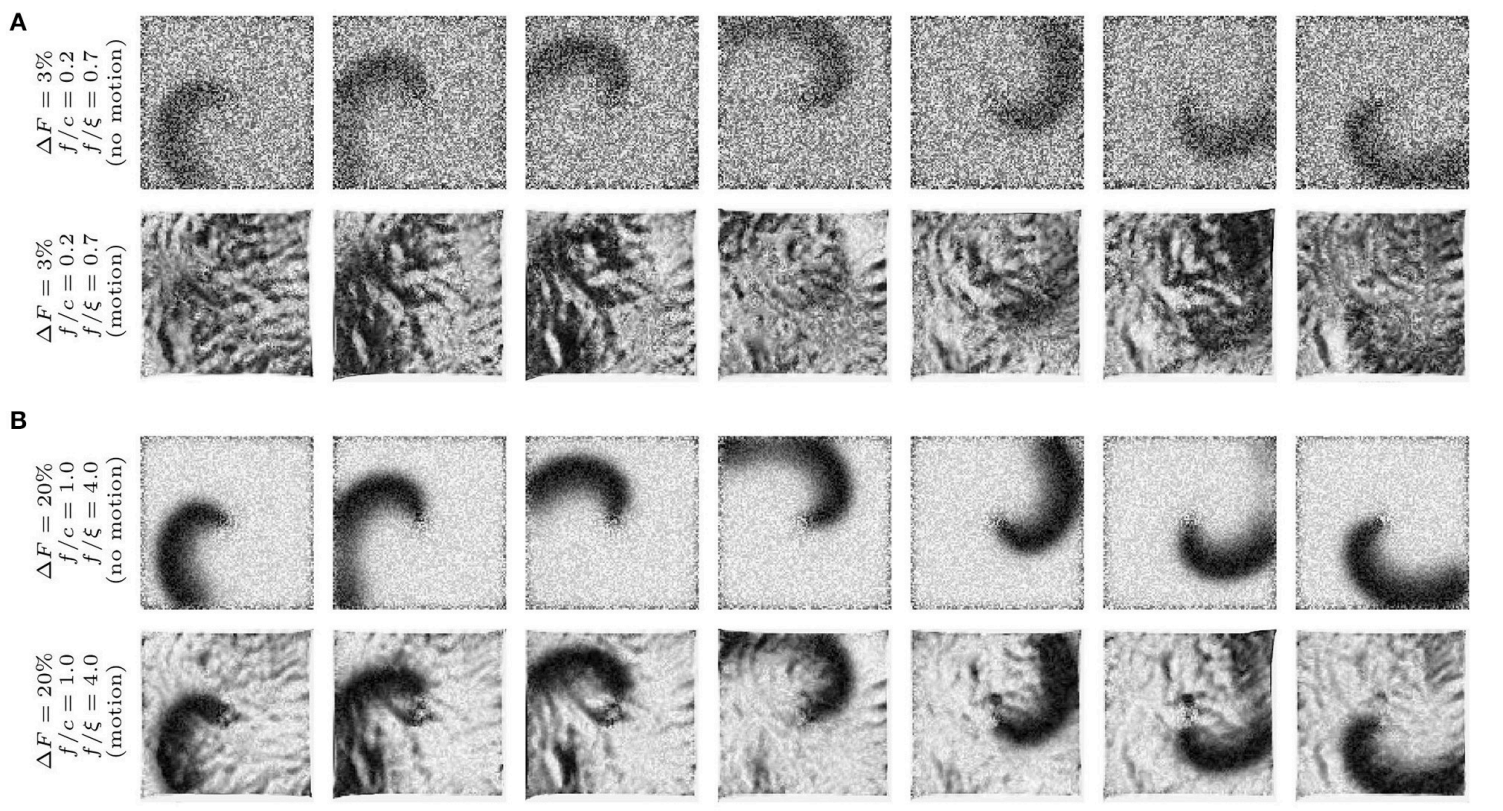
Synthetic optical mapping videos (pixel-wise normalized) with simulated spiral waves on a deforming heart surface. The spiral wave pattern is caused by electrical activity and displayed as an intensity decrease which is typically seen during voltage-sensitive optical mapping. Clockwise rotating spiral wave with two different signal strengths *f*: **(A)** weak signal (Δ*F*/*F* = 3%) and **(B)** strong signal (Δ*F*/*F* = 20%) on non-deforming (top sequence) and deforming (bottom sequence) heart surface. The noise ξ is constant in all image sequences (ξ = 0.03) even though it appears to be stronger in **(A)** due to the pixel-wise normalization (pixel intensities *I* ∈ [0, 1] dimensionless normalized units, n.u.). The amplitude of motion is the same in all image sequences ($|\vec{u}|\approx 3-5$ pixels). On the deforming surface the spiral wave pattern is superimposed by motion artifacts. **(B)** Due to the large signal strength *f*, the spiral wave is still visible on the deforming medium and motion artifacts are comparatively low. The amount of motion artifacts depends on the signal-to-contrast ratio *f*_*c*_ = |*f*|/*c*, the ratio of fluorescence signal strength *f* to the strength of the local contrast *c* or short-scale intensity gradients in the image (*c* constant in all images).

The electrical part of the model was simulated using the phenomenological, two-variable Aliev-Panfilov model (Aliev and Panfilov, 1996) comprised of two coupled partial differential equations with dynamic state variables *u* and *v*:

(1) $$\begin{array}{lll} \overset{\cdot }{u} & = & \nabla^{2}u-ku\left(u-a\right)\left(u-1\right)-uv \end{array}$$

(2) $$\begin{array}{lll} \overset{\cdot }{v} & = & \varepsilon \left(u\right)\left(ku-v\right) \end{array}$$

where *u* and *v* are dimensionless normalized representations of the transmembrane potential and the conductance of a slow repolarizing current, or excitatory and refractory dynamics, respectively. Note that the range of *u* is within the interval [0,1]. The value of *u* is used to modulate the video images, see section 2.2.2. The diffusion term ∇^2^*u* provides the diffusive coupling between neighboring cells of the electrical lattice and leads to spreading waves of electrical excitation through the excitable medium. *k*, *a*, and ε(*u*) are model parameters. The generation of active stress *T*_*a*_ due to excitation is modeled using a third partial differential equation that depends on the excitatory variable *u* as described previously (Nash and Panfilov, 2004):

(3) $$\begin{array}{lll} {\overset{\cdot }{T}}_{a} & = & \varepsilon \left(u\right)\left(k_{T}u-v\right) \end{array}$$

The equation simulates immediate and homogeneous active stress generation in response to electrical stimulation and simulates excitation-contraction coupling (Bers, 2002). The parameter *k*_*T*_ determines the magnitude of the active stress build-up in each cell of the model and defines the strength of the contractions occurring in the medium, see Figure 2E.

The elasto-mechanical model consists of a mass-spring damper system with controllable, tunable linearly transverse muscle fiber anisotropy (Bourguignon and Cani, 2000; Christoph, 2015). Figure 2B illustrates the lattice structure of the mass-spring damper system. The system consists of a regular lattice with cells defined by four vertices of the lattice and the cells containing sets of perpendicular springs attached to the barycenter and to the edges of the cells. The sets of springs can be oriented arbitrarily in the two-dimensional plane and introduce preferred orientations and anisotropy to the elastic system. One of the springs is set to be the active spring along which contractions occur upon electrical excitation, representing the fiber orientation. The springs rest lengths are modulated by the active stress variable *T*_*a*_, which is in turn dependent on the excitation *u*, the active stress inducing a shortening of the active springs, which results in the contraction of the cell. As a result, the tissue exhibits contractions and large deformations with length changes in the order of up to 10%. The cells at the boundaries of the elastic medium are connected to additional springs, which are fixed with one of their ends in space, mimicking an elastic interface with its surrounding.

Each cell of the elastic model corresponds to one cell of the electrical model. The model was solved using finite differences numerical integration schemes. The electrical model was solved using forward Euler integration and the elastic model was solved using Verlet integration. During integration, both models were updated simultaneously.

Figure 2E shows how the contraction strength and overall amount of deformation of the model can be varied with the parameter *k*_*T*_ from Equation (3). Typical values for the parameter *k*_*T*_ used in this study were *k*_*T*_ = [0.001, 0.005, 0.01, 0.1, 0.2, …, 3.0]. The graph shows that magnitudes of the displacements $\left|\vec{u}\right|$ exhibited by the nodes of the simulation grow rapidly with increasing *k*_*T*_ for very small *k*_*T*_ and less rapidly for larger *k*_*T*_. The graph shows the average displacements $<|\vec{u}|>$, which were computed from the maximal separation of the positions $\left|{\vec{x}}_{xy}\left(t\right)-{\vec{x}}_{xy}\left(t^{\prime }\right)\right|$ of one vertex (*x, y*) of the simulation grid over the entire time course of the simulation and averaging over all vertices.

#### 2.2.2. Synthetic video generation

The electrical patterns and deformations exhibited by the numerical model were used to create videos showing a deforming grayscale texture image, being locally superimposed by intensity modulations in locations where the tissue is electrically activated, see Figure 2. The video image is a video frame from one of the recordings obtained during the optical mapping experiments. The size of the video image of 100 × 100 pixels (slightly cropped) matches the grid size of the simulation. Therefore, in terms of spatial units, one cellular unit of the simulation domain corresponds to one pixel in the video data. Figure 2E correspondingly shows by how many pixels (approximately) the nodes of the mechanical grid move through the image plane (0.5 − 5.0 pixels). We found that for the chosen values for the parameter *k*_*T*_ we obtained similar magnitudes of motion as seen during the experiments. The intensity values of the video image were normalized *I* ∈ [0, 1] (dark cropped areas corresponding to values < 0.1).

First, the simulation output of the electrical model was used to create videos showing an optical mapping grayscale image of the heart surface superimposed by a rotating spiral wave pattern, the pixels' intensities decreased by a fraction of the value of the excitatory variable *u*, as shown in Figure 2A. Because each pixel of the original undeformed video image corresponds to a discrete cell of the simulation, there is a one-to-one correspondence between the pixel's original intensity value and the cell's value for *u*. More specifically, the time-varying two-dimensional maps of the electrical variable *u*(*x, y, t*) were used to decrease the otherwise static pixel intensity of the texture image *I*_*texture*_(*x, y*). The intensity value *I* in each pixel (*x, y*) was modulated linearly as follows:

(4) $$\begin{array}{lll} \tilde{I}\left(x,y,t\right) & = & I_{texture}\left(x,y\right)+f\cdot u\left(x,y,t\right)+\xi \end{array}$$

where |*f*| ∈ [0, 1] is a scaling factor and represents the maximal intensity change of the fluorescence-induced intensity modulations or fractional change in fluorescence in normalized units and ξ corresponds to noise. Typical values used in this study for the parameters were *f* = [−0.01, −0.03, −0.06, −0.12] and ξ = [0.005, 0.01, 0.03]. While in this study we only simulated a decrease in the signal with *f* < 0, mimicking the behavior of typical voltage-sensitive dyes, it would also be possible to simulate an increase in fluorescence with *f* > 0 as seen during calcium-sensitive imaging. The normalized fractional change in fluorescence *f* represents the fractional change in fluorescence Δ*F*/*F* exhibited by fluorescent dyes in optical mapping experiments. The texture image *I*_*texture*_(*x, y*) was normalized with all its intensity values in the range *I* ∈ [0, 1]. The histogram of grayvalues ρ(*I*) of the texture image could be scaled such that the image properties fulfilled specific criteria; for instance, the video images contrast matching a predefined local image contrast *c* with its corresponding contrast distribution, see **Figure 8C** and section 3.2.

The image frames of the resulting video sequence Ĩ(*x, y, t*) were then deformed within the two-dimensional image plane according to the deformed geometry or time-varying mechanical configuration χ(*t*) of the simulation grid, adding the displacements $\vec{u}\left(x,y,t\right)$ to the vertices defining each pixel, yielding a deformed, intensity modulated video Ĩ_χ_(*x, y, t*), see Figure 2C. The magnitudes of the deformations could be tuned using the parameter *k*_*T*_ from Equation (3). The average displacement strengths given in pixels achieved for various values for *k*_*T*_ are shown in Figure 2E. To obtain videos showing the moving, deformed texture Ĩ(*x, y, t*) in a static laboratory camera view frame, the video data was then resampled, redistributing the pixel intensity values of Ĩ(*x, y, t*) into a regular undeformed pixel grid, yielding resampled video sequences *I*(*x, y, t*), see Figure 2D. Resampling was performed using polygon clipping algorithms. The simulations typically included *N*_*t*_ = 50, 000 time steps, from which every 10*th* time step was extracted, the resulting image sequences then consisting of 5, 000 frames, showing about 10 spiral rotations.

### 2.3. Motion tracking

**Figure 4:**
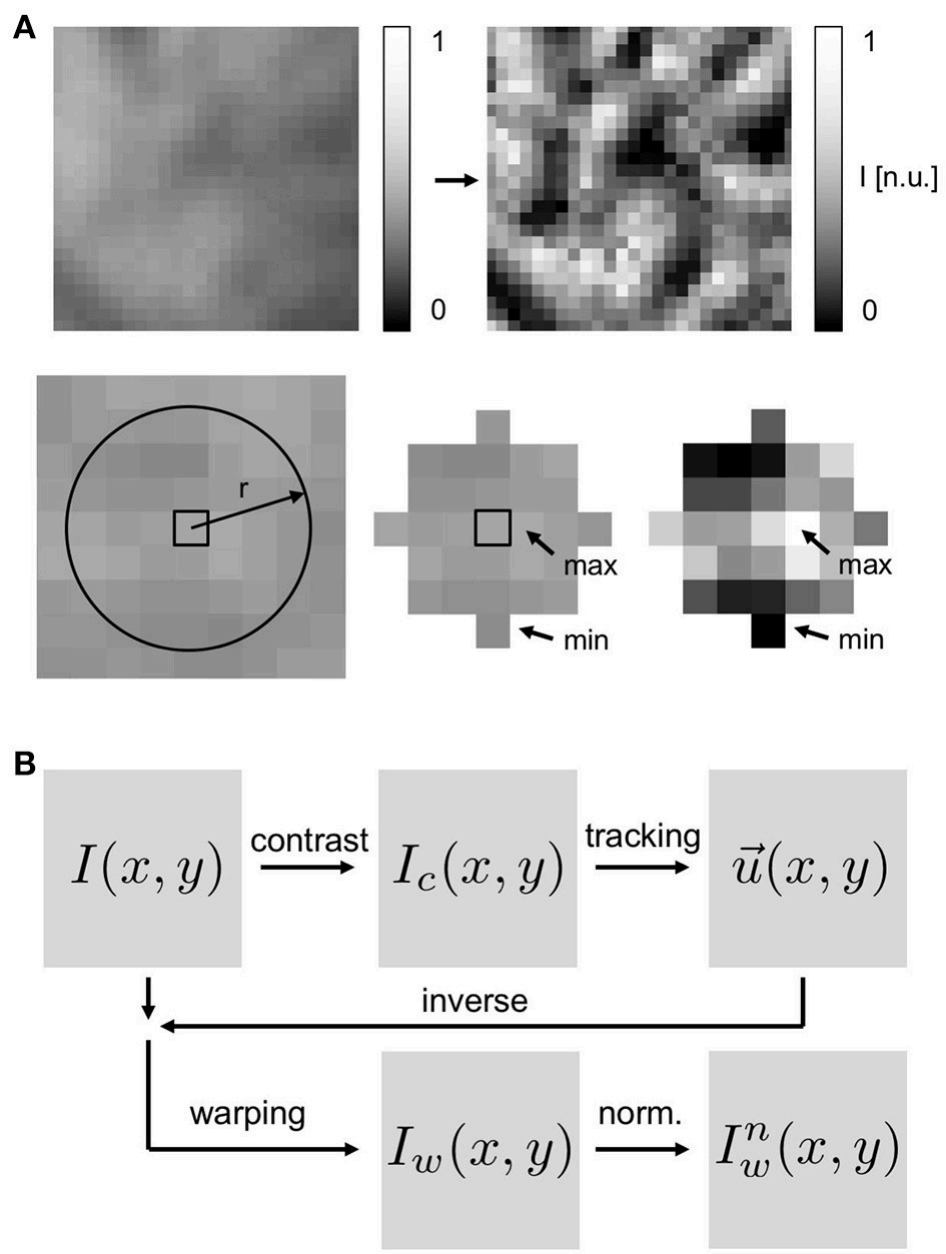
Motion tracking using contrast-enhanced video images: **(A)** Top left image: original, raw video image with pixel intensities normalized to minimal and maximal values in entire video (*I*(*x, y*) ∈ [0, 1]). Top right image: contrast-enhanced video image used for motion tracking (*I*_*c*_(*x, y*) ∈ [0, 1]) with pixel intensities normalized by minimal and maximal value in local neighborhood (disk-shaped region $S_{xy}$) around each pixel. Resulting contrast-enhanced images show maximally intensified short-scale image gradients and suppressed large-scale image gradients. **(B)** Schematic of motion tracking procedure with tracking of contrast-enhanced images *I*_*c*_. The original video images *I* are deformed using the inverse tracking data $-\vec{u}$ to obtain motion-stabilized or warped videos *I*_*w*_(*x, y, t*), which are then further post-processed, i.e., pixel-wise normalized ($I_{w}^{n}$).

Motion tracking was performed with both the experimental and the synthetic optical mapping videos to obtain two-dimensional in-plane displacement vector fields $\vec{u}\left(x,y,t\right)$, the displacement vectors indicating planar local shifts of tissue segments in the video images. Motion tracking was performed using a Lucas-Kanade optical flow estimation algorithm (Periaswamy et al., 2000, see also Christoph et al., 2017, 2018). Generally, the motion tracking algorithm is able to track rigid and non-rigid body motion, affine deformations as well as translational and rotational motion in images at a sub-pixel resolution. The motion tracking algorithm does not require any visible characteristic features, landmarks or markers attached to the heart surface to facilitate or assist the motion tracking. Instead it estimates optical flow that occurs in between two images. We found that simply the visible anatomical texture of the heart surface is sufficient to associate two local tissue segments in between two frames with each other. Nevertheless, we enhanced the anatomic texture and the visible features on the heart surface numerically to increase the accuracy and robustness of the motion tracking algorithm, see Figure 4 and section 3.3.

When tracking experimental data, short video sequences with durations of 1.0 − 10.0*s* (500 − 5, 000 frames, 500*Hz*) of the optical mapping recordings were extracted and stored as normalized videos containing intensity values *I* ∈ [0, 1] with floating point precision, the intensity values normalized by the maximal and minimal values found in the entire video sequence *v*_*s*_, see also Figure 1B:

(5) $$\begin{array}{l} I\left(x,y,t\right)=\left(v_{s}\left(x,y,t\right)-\min\left(v_{s}\right)\right)/\left(\max\left(v_{s}\right)-\min\left(v_{s}\right)\right) \end{array}$$

such that the experimental and the synthetic video data were stored in the same format. Next, in both the experimental and the synthetic data, the motion was tracked throughout the sequence of video images, comparing each video frame to one predefined reference frame *I*_*r*_(*x, y, t*) out of the sequence (*t* ∈ [1, …, *N*] frames), registering the shifts of the tissue in between the two frames in each pixel. For data showing periodic cardiac activity, a reference frame showing the undeformed, non-contracted heart shortly before (25*ms* ± 5*ms*) the electrical activation (depolarization) of the tissue was selected. For data showing arrhythmic cardiac activity, an arbitrary frame or a frame in the middle of the image sequence was selected as the reference frame. The motion was tracked either in the original normalized videos *I*(*x, y, t*), as derived in Equation (5), or in contrast-enhanced videos *I*_*c*_(*x, y, t*), see below, which show the maximally intensified contrast of the tissue, see Figure 4. To obtain contrast-enhanced videos, each pixel's intensity value *I*(*x, y, t*) was renormalized by the maximal and minimal intensity values found within a small disk-shaped sub-region $S_{x,y}$ around the pixel:

(6) $$\begin{array}{l} I_{c}\left(x,y,t\right)=\left(I\left(x,y,t\right)-\min\left(S\right)\right)/\left(\max\left(S\right)-\min\left(S\right)\right) \end{array}$$

the sub-region $S_{x,y}$ typically retaining a diameter of 5–7 pixels and renormalizing all pixels in each video frame individually. The conversion produced video sequences, in which the local tissue contrast is maximally intensified and the tissues' features and its unique local texture become very pronounced, see Figure 4A. The conversion also caused larger-scale intensity gradients across the images to vanish. Motion was then tracked in either the resulting contrast-enhanced videos *I*_*c*_(*x, y, t*) or the original simply normalized videos *I*(*x, y, t*) to compare the different outputs. The typical frequency of the detectable features in the video images is a few pixels (5–10), see section 3.2, and given by the granular, tile-shaped texture of the tissue. Two-dimensional in-plane displacements $\vec{u}\left(x,y,t\right)\in {\mathbb{R}}^{2}$ were determined for each pixel (*x, y*) in every frame *I*(*x, y, t*) or *I*_*c*_(*x, y, t*) throughout the normal or contrast-enhanced video image sequences. The displacement fields $\vec{u}\left(x,y,t\right)$ were stored for further analysis. Video data in which the heart deformed excessively or rotated, such that parts of the heart turned away from the camera or moved out of its field of view was discarded. Motion tracking (Matlab), warping and resampling (custom C++ code) and other processing requires approximately 1–3 min of computation time per video image (in the order 100 × 100 pixels) on a single CPU.

### 2.4. Motion stabilization and motion artifact removal

Using the displacement data obtained during the motion tracking procedure, we processed the original videos and produced warped or motion-stabilized videos in which motion appears to be absent or significantly reduced, see also Supplementary Video 3. The tracked displacements were used to deform each video image to match the image in the reference frame. More precisely, motion-stabilized or warped video images *I*_*w*_(*x, y*) were obtained by deforming the original video images *I*(*x, y*) using the inverse tracked displacements $-\vec{u}\left(x,y\right)$ to shift and deform each pixel accordingly, see Figure 4B. The deformed video image was then resampled in the image plane using the regular cartesian pixel grid yielding a deformed, resampled and motion-stabilized video image *I*_*w*_(*x, y*), see Figures 2D, 4B. The resulting frames showed a similarly deformed tissue configuration χ_*r*_ as shown in the reference frame *I*_*r*_ throughout the sequence of video images. For synthetic video data containing motion, the successfully tracked and warped video images *I*_*w*_(*x, y, t*) were very similar to the original undeformed video images Ĩ(*x, y, t*) containing only fluorescent activity.

### 2.5. Post-processing

Post-processing for the visualization of electrical waves was performed equally for both experimental and synthetic optical mapping data. Experimental video data was stored as unsigned (16 bit) integer valued-data and converted into floating-point valued data after normalizing each pixel by the minimal and maximal pixel value in the entire video, yielding normalized dimensionless pixel intensity values *I*(*x, y, t*) ∈ [0, 1] as described by Equation (5). The simulation data was stored as normalized data with all pixel values normalized *I*(*x, y, t*) ∈ [0, 1]. To visualize wave activity the video data was normalized pixel-wise, meaning that each time-series *I*_*xy*_(*t*) in each pixel was normalized individually by its minimal *I*_min_ = min(*I*_*xy*_(*t*)) and maximal *I*_max_ = max(*I*_*xy*_(*t*)) intensity value respectively, see also (Laughner et al., 2012):

(7) $$\begin{array}{l} I_{n}\left(x,y,t\right)=\left(I\left(x,y,t\right)-I_{\min}\right)/\left(I_{\max}-I_{\min}\right) \end{array}$$

Alternatively, the video data was normalized pixel-wise using a sliding-window normalization, meaning that each time-series *I*_*xy*_(*t*) in each pixel was normalized individually by the minimal *I*_min_ = min(*I*_*xy*_(*t*)) and maximal *I*_max_ = max(*I*_*xy*_(*t*)) intensity value found within a temporal window of size *t* ± *w*, respectively, the window size being at least one cycle length of the wave activity (e.g., approx. 100*ms* during ventricular fibrillation). Both normalizations equally amplify temporal intensity fluctuations in each pixel (cf. **Figure 7**) and they are typically used to visualize action potentials or calcium cycling. However, both normalizations can also amplify intensity fluctuations that are not produced by electrical activity but instead by optical flow or in other words motion artifacts.

In the synthetic data, motion artifact strengths $\tilde{m}\left(x,y,t\right)$ were computed by calculating the absolute difference between the motion-stabilized video, which was obtained in an exact co-moving frame, and the video obtained in a static camera view or lab frame before or after tracking. The exact co-moving video corresponds to an idealized measurement, in which the amplified, normalized fluorescent signal can be measured precisely in each tissue coordinate. This video is not available in experiments because motion tracking algorithms are imperfect and the tracked tissue configuration is only an approximation of the real tissue configuration. In simulations, however, the real tissue configuration is available. Motion artifact strengths $\tilde{m}$ were computed using the pixel-wise normalized videos:

(8) $$\begin{array}{l} \tilde{m}=\underset{x,y,t}{\sum }\left(\left|{\tilde{I}}_{n}\left(x,y,t\right)|-|I_{n}\left(x,y,t\right)\right|\right) \end{array}$$

summing the absolute differences of the two videos' pixel intensities over all pixels and video frames. The video Ĩ_*n*_(*x, y, t*) that was obtained in the exact co-moving frame was first pixel-wise normalized, then deformed and finally resampled. The video *I*_*n*_(*x, y, t*) that was obtained in a static camera view frame with pixel-based video-processing was first deformed, then resampled and finally pixel-wise normalized. The first video will never contain motion artifacts and the latter video may contain motion artifacts.

## 3. Results

### 3.1. Efficacy of motion artifact removal in experimental data

**Figure 5:**
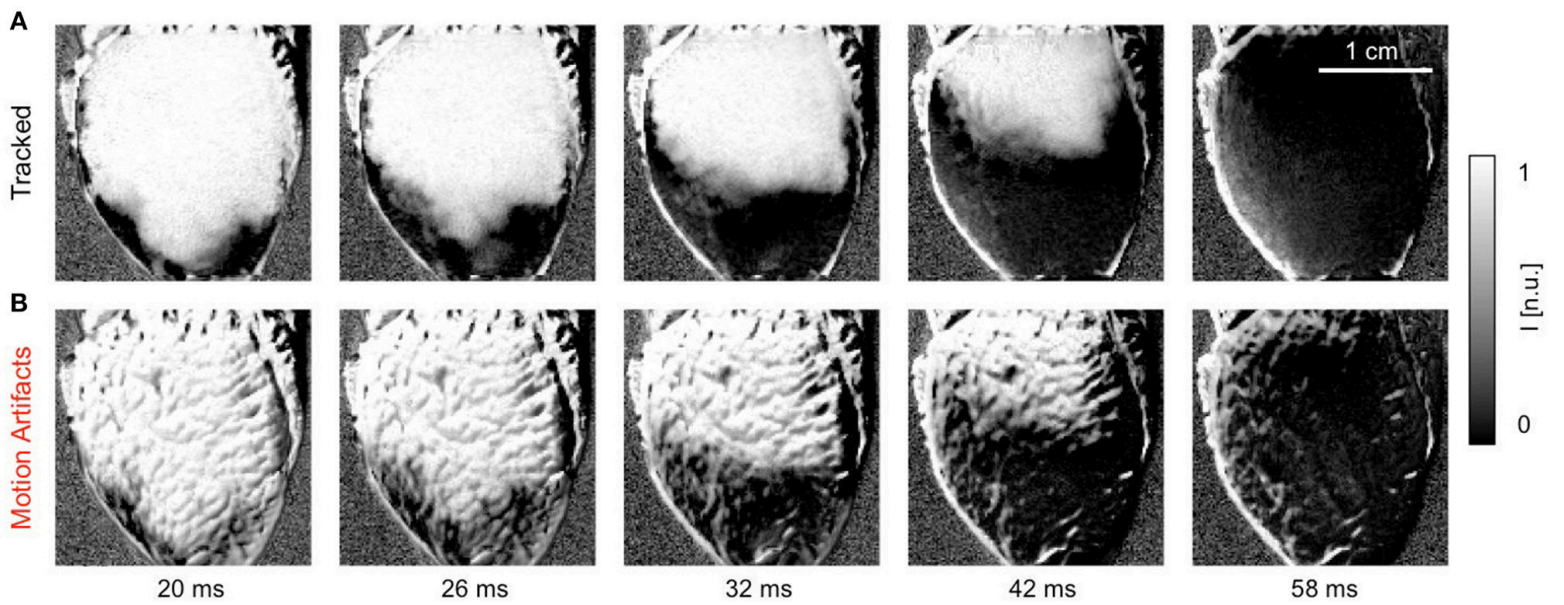
Efficacy of motion tracking and motion artifact compensation during electromechanical optical mapping: planar action potential wave propagating upwards across ventricular surface of a rabbit heart as the heart contracts (dark areas correspond to depolarized tissue and bright areas correspond to undepolarized tissue, staining Di-4-ANEPPS, pixel-wise sliding-window normalization [n.u.]). See also Supplementary Video 2. **(A)** Motion-stabilized image sequence with substantial reduction in dissociation-related motion artifacts after motion tracking and numerical motion-stabilization. **(B)** The same image sequence without motion tracking and stabilization. Motion artifacts appear as a high-frequency, network-like spatial pattern with black-and-white deflections superimposing and distorting the action potential wave pattern. Both image series were processed in the same way before and after motion tracking.

Figure 5 shows a quasi-planar action potential wave propagating across the contracting ventricular surface of a rabbit heart, see also Supplementary Video 2. The wave was elicited after the application of a pacing stimulus on the endocardial wall close to the apex of the heart using a MAP-catheter electrode. As a result, the wave propagated upwards from the apex toward the base of the heart. Due to the voltage-sensitive staining and long-pass emission filtering, the fluorescent intensity decreases on the detector during the depolarization of the action potential. Correspondingly, activated or depolarized tissue corresponds to dark regions, whereas undepolarized tissue during the diastolic interval corresponds to bright regions in the image. The video data was normalized using a pixel-wise sliding-window normalization, see section 2.5. Other post-processing such as smoothing was not performed. The upper image series shows the action potential wave visualized after tracking and motion stabilization in the co-moving frame, in which motion artifacts appear to be absent or at least substantially reduced. The action potential propagates across the ventricular surface as the heart contracts. Depolarized and undepolarized tissue areas correspond to two clearly distinguishable, homogeneous areas with low and high light intensities due to the pixel-wise normalization of the data, see Equation (5). For comparison, the lower image series shows the same image sequence without motion tracking and motion stabilization. Due to the shifts of the tissue and a deallocation or dissociation of tissue regions and their corresponding pixel on the camera sensor measuring the tissue region during this pixel-based measurement, the video contains dissociation-related motion artifacts. Such dissociation motion artifacts appear as a network-like, tile-shaped spatial pattern with black-and-white deflections superimposing and distorting the action potential wave pattern, which can be seen in the upper image sequence. Note that both image series were processed in the same way, normalizing the optical traces obtained in each pixel using a sliding-window normalization, see Equation (5). However, the motion that is still present in the lower image series causes the undesired high-frequency spatial motion artifact patterns. In contrast, because the motion was tracked and stabilized in the upper image sequence before the post-processing, the high-frequency spatial motion artifact pattern vanished.

**Figure 6:**
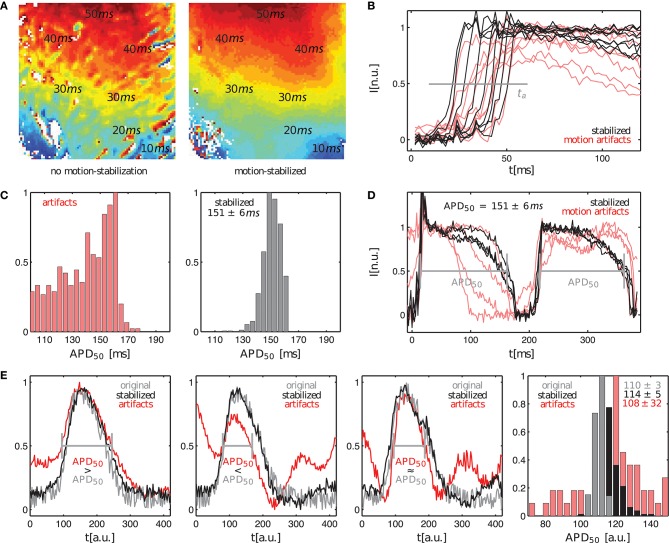
Comparison of activation time and action potential duration measurements in optical maps with and without motion artifacts. **(A)** Activation maps showing quasi-planar action potential wave propagation on left ventricular surface of contracting rabbit heart before tracking and without motion-stabilization (left) and after tracking and with motion-stabilization and motion artifact compensation (right). **(B)** Upstroke detection (at 0.5) for computation of activation maps in **(A)**. **(C,D)** Action potential duration measurements of < *APD*_50_ > = 151*ms* ± 6*ms* for motion-stabilized video data. Variance in action potential duration due to motion decreases with motion compensation. **(E)** Comparison of uncertainty in action potential duration measurements in original artifact-free data (APD_50_ = 110 ± 3*a*.*u*.) and tracked, motion-stabilized synthetic video data (APD_50_ = 114 ± 5*a*.*u*.). Uncertainties without motion or motion compensation are both σ_*APD*_ < 5% and are much larger with motion.

Further analysis of the motion-stabilized video data shows that it is possible to improve the robustness and accuracy of measurements, such as activation time or action potential duration measurements. Figure 6A shows activation maps computed for the quasi-planar action potential wave shown in Figure 5. The activation map on the left was computed from the raw, non-tracked video data including motion artifacts. The activation map on the right was computed from the tracked, motion-stabilized video data. Both maps exhibit a global gradient from short (blue: *t*^*a*^ < 10*ms*) to long activation times (red: *t*^*a*^ > 40*ms*), beginning with short activation times close to the apex and large activation times further up on the ventricular surface. The gradients in each of the activation maps reflect the situation in Figure 5, in which a wave travels across the ventricular wall starting from the apex toward the base of the heart. However, one can observe strong artifacts in the raw activation map containing motion. In about 15% of the pixels the activation times deviate strongly (>10%) from the activation times in the tracked map. It seems that the motion artifacts seen in the normalized video sequence (lower sequence) in Figure 5 similarly manifest in the activation maps. Otherwise, the high similarity of both activation maps suggests that activation times and activation maps can, at least to a certain extent, be computed from unprocessed video data. The maximal activation time, that is the time which was required for the wave to traverse the entire field of view, is equal in both untracked and tracked maps with $t_{max}^{a}=52ms\pm 2ms$. We frequently found that in the uncompensated, raw video data with large fluorescent signals (here Δ*F*/*F* ≈ 6 − 8%) and moderate motion, simply the upstroke of the action potential can be sufficient for the computation of activation times, provided it can be detected appropriately. However, note that without motion tracking, the measurement of activation times is nevertheless inaccurate because in a non-co-moving frame of reference the spatial correspondence is lost. Figure 6B shows exemplary traces of the action potential upstrokes obtained from both the raw and the motion-compensated videos for comparison. In the uncompensated optical maps, the action potential upstrokes are in some cases not as steep or pronounced as in the motion-stabilized optical maps. This is the probable cause of the artifact patterns seen in the raw activation map (left) in Figure 6A.

Figures 6C,D shows action potential duration measurements obtained from the same data set shown in Figure 5. The action potential duration (APD) of the 2 subsequent action potentials shown in Figure 6B was measured in approximately 500 traces obtained from the tracked, motion-stabilized video data by computing the delay between the upstroke and repolarization times at 50% of the height of the action potentials (APD_50_), or at a value of 0.5 in the normalized videos. Figure 6C shows histograms with the distributions of action potential durations for the raw, untracked video data including motion artifacts and the tracked, motion-stabilized video data. In case of the raw data without motion-stabilization, the histogram exhibits a broad distribution of APDs, which prohibits the unique identification of a dominant action potential duration. With motion-stabilization, the histogram exhibits a narrow distribution from which it is possible to determine a mean action potential duration of *APD*_50_ = 151 ± 6*ms*. A similar drastic decrease in APD measurement uncertainty after tracking is also found by Khwaounjoo et al. (2015).

Figure 6E shows the variability and uncertainty in the action potential duration measurement in synthetic video data with motion. Three exemplary plots obtained from three adjacent sites show the original artifact-free course of the simulated action potential (gray) together with the tracked, motion-stablized curves (black) and the curves including motion-artifacts (red) for average maximal displacements of $<|{\vec{u}}_{max}|>=3.2$ pixels. The original and the motion-stabilized curves (gray and black) can barely be distinguished from each other given the pronounced amount of noise, while the curves including motion artifacts (red) deviate strongly from both the original and motion-stabilized curves. The right panel in Figure 6E shows the histogram with the three respective distributions of action potential durations (computed with the video data smoothed with kernel sizes *k*_*x*_ = *k*_*y*_ = 3 pixels and *k*_*t*_ = 11 time steps; the smoothing provides more robust upstroke and repolarization time detections). As for the experimental data, the distribution of measured action potential durations with motion artifacts is very broad with a high variability in action potential durations. The histogram's maximum indicates an APD_50_ ≈ 120*a*.*u*., which deviates by about 17% from the actual value. The average (APD_50_ = 108 ± 32*a*.*u*.) only deviates by about 2% from the true value, but the distribution is very broad and has a large uncertainty of 30%. Both the original and the motion-stabilized distributions are narrow and very distinctly exhibit a peak, which we used to compute the average action potential durations of APD_50_ = 110 ± 3*a*.*u*. and APD_50_ = 114 ± 5*a*.*u*., respectively. The tracked, motion-stabilized data deviates by 3.6% from the original data and exhibits a slightly higher uncertainty of σ_*APD*_ = 4.5% (about 1.7 times as large as the uncertainty for the original data with σ_*APD*_ = 2.7%).

**Figure 7:**
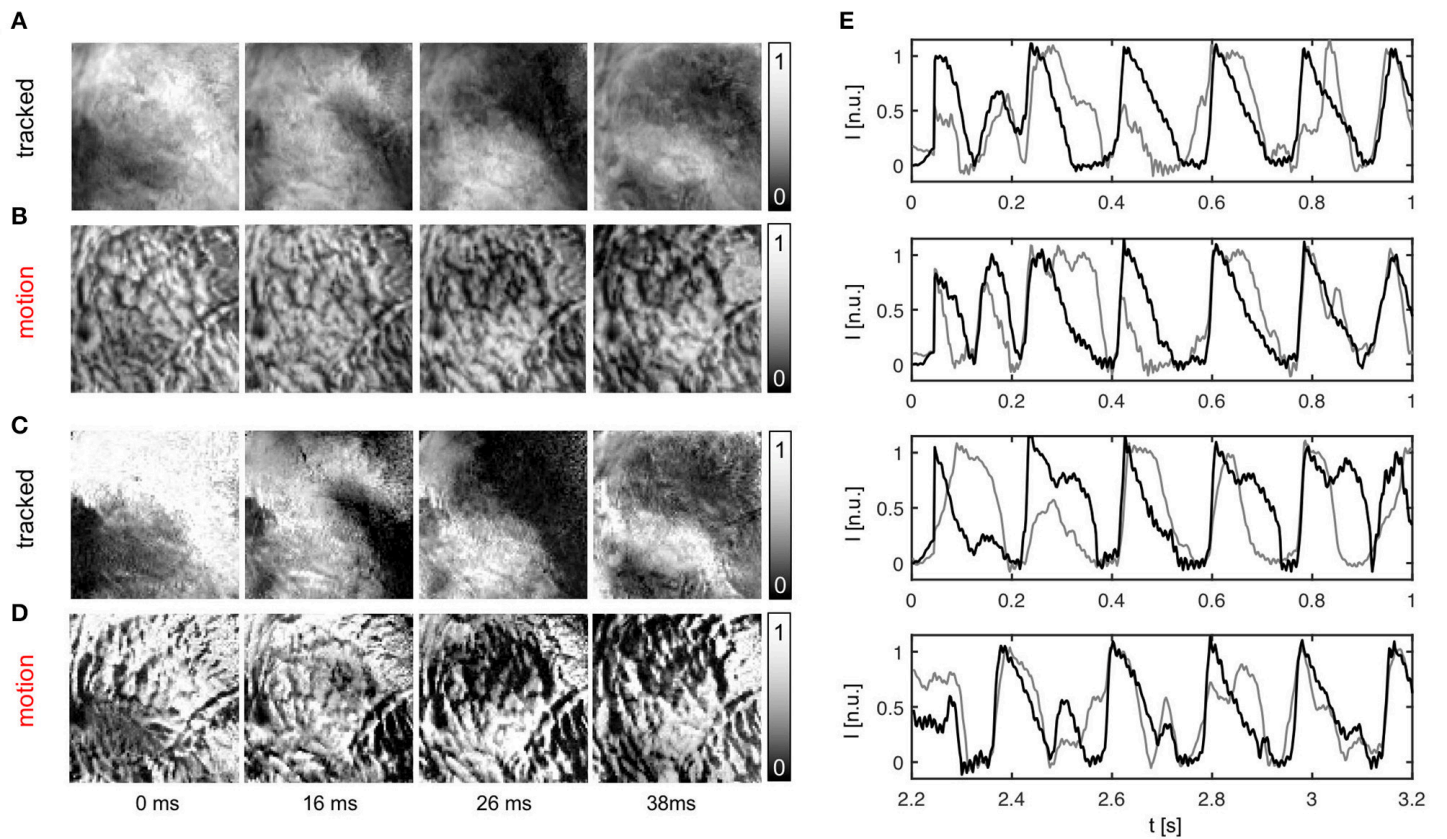
Action potential vortex waves mapped on contracting left ventricular surface of rabbit heart during ventricular fibrillation. Electromechanical optical mapping was performed with voltage-sensitive staining (Di-4-ANEPPS, 128 × 128 pixels, 500 fps, approx. 1 × 1 cm field of view). **(A,C)** Maps with substantially inhibited motion artifacts after tracking and motion-stabilization showing action potential waves. **(B,D)** Action potential wave maps without tracking and motion stabilization. Maps exhibit strong motion artifacts. In **(A,B)** and **(C,D)** the same activity is shown with two different normalizations, see main text. **(E)** Exemplary time-series obtained from optical maps with motion before (gray) and after tracking and numerical motion-stabilization (black).

The efficacy of the tracking algorithm in inhibiting motion artifacts during arrhythmias is demonstrated in Figure 7. The image sequences in Figure 7A show chaotic action potential vortex wave activity mapped on the contracting rabbit heart surface during ventricular fibrillation. Just as in Figure 5, motion artifact patterns have decreased drastically due to the motion tracking. Instead of heavy motion artifacts (B,D) one immediately observes action potential wave patterns (A,C). Again, due to the voltage-sensitive imaging, depolarized tissue corresponds to dark and repolarized or inactivated tissue to white areas. For comparison, we show the same data for two different normalizations [A,B: pixel-wise as in Equation (5); C,D: pixel-wise within sliding-window with τ = 140 − 160*ms*]. The two different normalizations show that the motion artifact pattern and the reduction in motion artifacts is independent from other processing steps and the particular visualization of the wave pattern. In both uncompensated optical maps that are obscured with motion artifacts one can observe the typical high-frequency spatial motion artifact patterns, as observed in Figure 5.

In summary, Figures 5–7 demonstrate that it is possible to perform optical mapping experiments with beating isolated hearts and to reliably retrieve optical maps with substantially reduced motion artifacts from the moving, contracting heart surface during regular and irregular cardiac rhythms. The differences between the raw and motion-stabilized video data are substantial and can immediately be identified in the optical maps. Motion artifacts correspond to high-frequency, short-scale spatial patterns, which are absent in the registered, co-moving maps.

### 3.2. Characterization and quantification of motion artifacts

**Figure 8:**
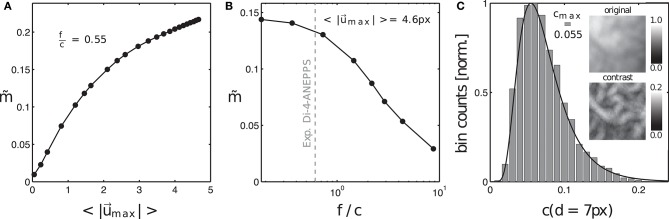
Motion artifact strength $\tilde{m}$ generated systematically in synthetic optical mapping videos, see also Figure 3. **(A)** Increase in motion artifact strength $\tilde{m}$ with increasing amplitudes of motion $\left|\vec{u}\right|$. **(B)** Decrease in motion artifact strength $\tilde{m}$ with increasing fluorescent signal strength *f* or decreasing local tissue contrast *c*. The strength of motion artifacts depends on the relative signal-to-contrast ratio *f*/*c*. **(C)** Distribution of image contrast in optical mapping video frame used to generate synthetic optical mapping video. Top image: original texture, normalized to [0, 1]. Bottom image: contrast image. Image contrast *c* is the maximal absolute intensity difference that is found around a pixel (*x, y*) within a disk-shaped region $S_{xy}$ with diameter *d* (here typically 5−7 pixels).

**Figure 9:**
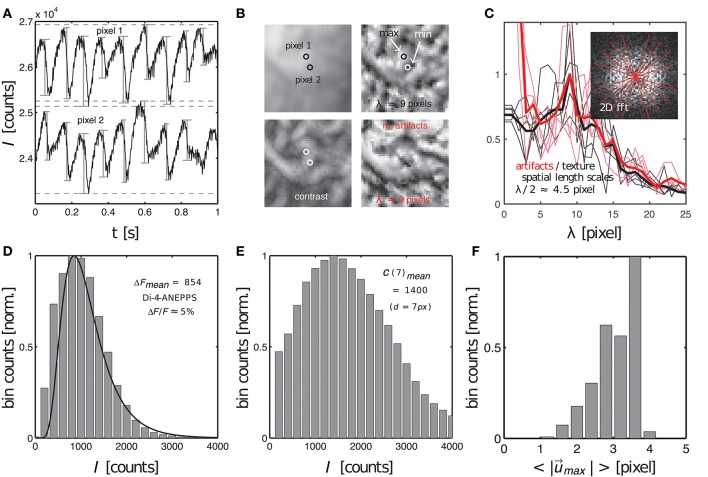
Characterization of dissociation-related motion artifacts in optical mapping videos showing the contracting, fluorescing heart surface. Measurements of important quantifiers: fluorescent signal strength *f*, image contrast *c*, amplitude of motion $\left|\vec{u}\right|$ and characteristic length scales λ of short-scale image gradients. **(A)** Action potentials measured during ventricular fibrillation in two close by pixels (see **B**) on the surface of a mildly contracting rabbit heart [graph reproduced from Christoph et al. (2017)]. Difference in baseline reflects different pixel intensities. **(B)** Raw optical mapping video image (top left), contrast image (bottom left), contrast image normalized to local minima and maxima (top right) and motion artifacts (bottom right). All images have the same characteristic texture with the same characteristic length scale or dominant spatial frequency (λ ≈ 9 pixels). **(C)** Radial profiles (fat lines: average) within 2D power spectra of spatial patterns (contrast image and motion artifacts) indicate dominant frequency of λ ≈ 9 pixels (see two overlapping peaks). **(D)** Fluorescent signal strength or fractional change in fluorescence Δ*F* (given as intensity counts *I*) measured from sequences of action potentials, see **(A)**, with the peak of the distribution at Δ*F* = 854 intensity counts in 16-bit video image. **(E)** Image contrast (given as intensity counts *I*) with peak at *C* = 1400 in 16-bit video image. **(F)** Amplitudes of motion during ventricular fibrillation (2 − 4 pixels, 128 × 128 pixel sensor size).

**Figure 10:**
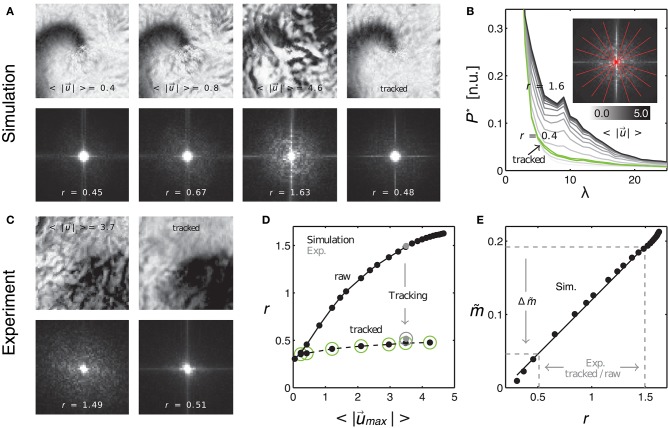
Evaluating tracking performance and residual motion artifacts by comparing synthetic to experimental data. **(A)** Synthetic optical maps before (panels 1–3) and after (panel 4) tracking. Increasing amplitudes of motion (panels 1–3: 0.4, 0.8, and 4.6 pixel) cause an increase in motion artifacts. Tracking and motion stabilization (panel 4) inhibits (residual) motion artifacts to levels found at 0.4–0.8 pixels of motion before tracking. Spectral maps obtained from the optical maps (bottom) show an increase in high-frequency spectral components with increasing motion. Tracking and motion compensation decreases the high-frequency spectral components to a level comparable to the left image (*r* = 0.45) with low amounts of motion (0.4 pixels). **(B)** Radial profiles of normalized spectral maps indicating an increase in width of the profiles (color-coded from gray to black, *r* = 0.4 to 1.6) with increasing motion and decrease in width after tracking (green curve). **(C)** Comparison with experimental data. Tracking and motion-stabilization reduces motion artifacts and radial profile width *r* (pre-tracking *r* = 1.49, post-tracking *r* = 0.51). **(D)** Profile width *r* over magnitude of motion $<|\vec{u}|>$ for raw and tracked (circles) simulated (black dots) and experimental data (gray dots). **(E)** Linear increase of motion artifact strength $\tilde{m}$ with spectral profile width *r*. Motion artifact reduction $\Delta \tilde{m}$ calculated for experimental data via the linear relationship between motion artifact strength (obtained in simulations mimicking experimental data) and spatial high-frequency components in optical maps (quantifiable by *r* with both experimental and simulation data).

Here, we introduce a framework for characterizing and quantifying motion artifacts, analyzing their spatial characteristics and appearance in optical maps. As can be seen in Figures 3, 5B, 7B,D, motion produces a very characteristic network-like, tile-shaped dark-bright spatial pattern of pixel intensities in normalized optical maps. We reproduced this characteristic motion artifact pattern in synthetic optical maps and systematically varied important video properties, such as displacement or contraction strength and fluorescent signal strength (Δ*F* or *f*), to determine their contribution to the emergence of this spatial motion artifact pattern. Next, we related the data to motion artifact patterns found in experiments, see Figures 8–**10**. Figure 8A shows how motion artifacts $\tilde{m}$ increase with increasing amplitudes of motion (here for given values of signal strength *f* and image contrast *c*, see below) in the synthetic data. The overall amplitude of motion is given as the average of maximal displacements $<|{\vec{u}}_{max}|>$, computed by averaging the magnitudes of the maximal shifts $\left|{\vec{u}}_{max}\left(x,y\right)|=\max|{\vec{x}}_{i}-{\vec{x}}_{j}\right|$ that each vertex (*x, y*) underwent throughout the simulation. The maximal shifts are the maximal distances measured in pixels between a vertex at time *t*_*i*_ and the same vertex at time *t*_*j*_. Motion artifacts occur even with slight tissue movement (finite and quickly increasing $\tilde{m}$ for < 1 pixel), which underscores the sensitivity of optical mapping to motion. The graph also shows that the strength of motion artifacts $\tilde{m}$ increases less quickly for shifts larger than ~3 − 5 pixels, indicating an involvement of other mechanisms in the emergence and development of motion artifacts, see Figure 8A. Next, Figures 8B,C show that the strength of motion artifacts does not only depend on the strength of the contraction and amplitude of the motion, but also depends on the signal strength *f*, as well as on the local image contrast *c* and their relative magnitudes with respect to each other. The signal strength *f* is the strength or amplitude of the fluorescent signal in the raw, normalized synthetic video data. In the experiments it would correspond to the fractional change in fluorescence Δ*F*/*F*. The local image contrast *c* is a measure for the maximal intensity differences that can be found in a small sub-region $S_{xy}$ around each pixel, see Equation (10). The image contrast *c* expresses the likeliness of such intensity differences to cause dissociation-related motion artifacts. Dissociation-related motion artifacts occur due to a loss of the sensor-tissue correspondence and are consequently produced by optical flow on the camera sensor caused by the tissue's movements through the video image. Considering these key determinants of motion artifacts, we created synthetic optical mapping videos for various values of the fluorescent signal strength *f*, the local image contrast *c* and varying amplitudes of motion $<|{\vec{u}}_{max}|>$. In the following, the fluorescent signal strength *f* and image contrast *c* are given as normalized units, where *f* ∈ [0, 1] represents a normalized unit of maximal fractional change in fluorescence intensity and *c* ∈ [0, 1] represents a normalized unit of image contrast. Figure 8B shows how motion artifacts remain small for large simulated fluorescent signals *f*, i.e., they are less noticeable, and increase with decreasing signal strength and saturate at a stationary value (~0.15) with vanishing signal *f*. More importantly, the graph demonstrates that increasing local image contrast *c* promotes the emergence of motion artifacts. With fixed fluorescent signal strength *f* and increasing image contrast *c*, i.e., the intensity of structures and features visible in the image, motion artifacts become larger. Motion artifacts are thus a relative measure. With little or no fluorescent signal (*f* = 0) or very large image contrast compared to the signal strength (*f*/*c* < < 1) one obtains in large parts only motion artifacts during an optical mapping measurement.

Accordingly, Figure 3 illustrates how motion artifacts emerge under various conditions in synthetically generated optical maps. In particular, it highlights how motion artifact strength $\tilde{m}$ varies greatly with differing signal strengths *f*. The image series show a clockwise rotating action potential spiral wave, which induces a dark spiral wave pattern on an otherwise brighter background in the synthetic video data. In real optical mapping experiments, such data would be obtained with voltage-sensitive staining (for instance using Di-4-ANEPPS). The strength of the motion is the same in all images. The video data was normalized pixel-wise, as described by Equation (5) and equally as shown in Figure 5A, to facilitate viewing of the intensity fluctuations caused by the electrical wave activity. The image series in Figure 3A show a spiral with low signal strength (*f* = −0.02) on a non-deforming vs. a deforming heart surface, respectively. The image series in Figure 3B show a spiral with larger signal strength (*f* = −0.12) on a non-deforming vs. a deforming heart surface, respectively. Due to the absence of motion in the upper image series in each of the two panels, the spiral wave patterns are not obscured by motion artifacts, they are artifact-free. In contrast, the same spiral wave patterns shown in the lower image series in each panel are obscured by motion artifacts. It is important to note that the noise level (ξ = 0.03) and the local image contrast (*c* = 0.055), as well as the amplitude of the contraction and motion ($<|{\vec{u}}_{max}|>\approx 3-5$ pixel), are the same in all four image sequences. Due to the pixel-wise normalization, which normalizes all activity including noise and eliminates differences in absolute signal strength (baseline), all relative signal intensity changes become amplified to the same level. Therefore, the spiral wave in the upper image sequence in Figure 3A is superimposed by stronger noise and is perceived weaker in comparison to the upper image sequence in Figure 3B, which contains a stronger signal in comparison to the noise level (ξ_1_ = ξ_2_ = 0.03). Comparing the two image series with motion shown in Figures 3A,B, one notices that the deformed image sequence with low signal strength *f* is heavily distorted and obscured by motion artifact patterns, while the deformed image sequence with high signal strength *f* is less affected by motion artifacts. The spiral wave pattern is nevertheless visible. Were the image contrast in the upper image sequence larger (*c*_1_ > *c*_2_) and the two signal strengths of both spirals the same (*f*_1_ = *f*_2_), one would obtain a very similar outcome. The figure illustrates that with increasing fluorescent signal strength *f* or decreasing image contrast *c* motion artifacts become less severe. Therefore, we suggest defining the signal-to-contrast ratio:

(9) $$\begin{array}{lll} f_{c} & = & \frac{f}{c} \end{array}$$

which indicates the relative signal strength *f* in comparison to the local image contrast *c* and gives an estimate for the likeliness of motion artifacts to be visible in optical mapping data. Both values *f* and *c* can be determined in experimental data, see Figures 9D,E and below. The situation in the image sequence shown in Figure 3A (*f*_*c*_ = |*f*|/*c* = 0.02/0.055 = 0.36) would be observed during voltage-sensitive imaging with Di-4-ANEPPS with fractional changes in fluorescence intensity typically ranging in the order of Δ*F*/*F* ≈ −3% to −8%. Strong signal strengths *f* as shown in the image sequence in Figure 3B (*f*_*c*_ = |*f*|/*c* = 0.12/0.055 = 2.2) are typically encountered, for instance, during calcium-sensitive imaging with Rhod2-AM with fractional changes in fluorescence intensity typically ranging in the order of Δ*F*/*F* ≈ 10 − 30%. Note that the synthetic videos in Figure 3 were generated by deforming a video image that was obtained in an optical mapping experiment with voltage-sensitive staining (Di-4-ANEPPS). The image shows the typical granular texture of the ventricular surface of a rabbit heart, which one similarly encounters with other fluorescent dyes (Rhod-2 AM, Di-4-ANBDQPQ) and other species.

The image contrast *c* in both the synthetic and experimental video images was determined to be the peak of distribution of local image contrasts, which were computed for every pixel (*x, y*) showing the heart surface in the raw, normalized video image, see Figure 9E. The histogram in Figure 9E shows the distribution of image contrasts computed for the original raw video image *I*(*x, y*) shown in the upper left subpanel of Figure 9B. This is the same image used to create the synthetic optical maps shown in Figure 3. The image in the lower left subpanel is the resulting contrast image *I*_*c*_(*x, y*). The image contrast *c* in each pixel (*x, y*) of that contrast image was computed within a small disk-shaped sub-region $S_{xy}$ around the pixel:

(10) $$\begin{array}{lll} I_{c}\left(x,y\right) & = & max\left(S_{d}\left(x,y\right)\right)-min\left(S_{d}\left(x,y\right)\right) \end{array}$$

The diameter *d* of the sub-region $S_{xy}$ was typically chosen to range in the order of *d* = 5 − 7 pixels. Note that while the raw video image *I*(*x, y*) contains values between *I* ∈ [0, 1], the contrast image *I*_*c*_(*x, y*) only contains values between approximately *I*_*c*_ ∈ [0, 0.2]. These are the magnitudes of image intensity differences that can typically be found within short length scales of a few pixels in the normalized and otherwise unprocessed video data. This length scale is significant because the amplitudes of the motion may occur on a similar length scale. In this case the intensity differences would create optical flow that may ultimately lead to motion artifacts, see below. How such local intensity gradients in the video images can easily obscure action potential signals is illustrated in Figure 9A, which shows two time-series of a sequence of action potentials measured on the ventricular surface of a moderately contracting rabbit heart during ventricular fibrillation (graph reproduced from Christoph et al., 2017). The two time-series were extracted from two nearby sites (pixel 1 and pixel 2) only a few pixels apart in the pixel plane and only a few hundred micrometers apart on the surface of the heart, see Figure 9B. Both time-series in Figure 9A possess different baselines, about 2, 000 intensity counts apart from each other, and exhibit slight motion artifacts, which become apparent as modulations of the traces around the baseline. The magnitude of the downstrokes (upstrokes of each action potential) are in the order of 1, 000 counts (Δ*F*), see Figure 9D, and are smaller than the difference in baseline. Hence, if both sites were to move toward each other or to switch their positions, the potential difference in baseline, i.e., the difference in local intensity or image contrast, could easily override the fluorescent signal Δ*F*, which is much smaller. The signal-to-contrast ratio in this recording is *f*_*c*_ < 1.

Next to the fluorescent signal and image contrast strengths, it is also important to consider the spatial length scales of image gradients and the magnitude of the displacements or motion seen in the video images. Figure 10B displays the original video image (top left) used in the simulations, its contrast image (bottom left) computed using the formula given in Equation (10), a contrast-enhanced locally normalized version of the original image (top right) computed using the formula given in Equation (8), and resulting motion artifacts appearing in this region without or with very little fluorescent signal *f*_*c*_ < < 1 (bottom right). One can see that all four images retain similar spatial frequency components. In particular, the image showing the local image contrast (lower left) and the image showing the motion artifacts (lower right) exhibit analogous spatial patterns with similar frequency components. The comparison illustrates how the loss of correspondence and dissociation in an optical mapping experiment leads to dissociation-related motion artifacts and links the phenomenon to the image contrast. Figure 10C shows that the dominant spatial frequency components measured from the two-dimensional Fourier-transforms of both the contrast-enhanced, locally normalized image (upper right) and the motion artifact patterns (lower right) in Figure 10B are equal at λ = 9 ± 1 pixels (peaks in red and black curves). This means that the distances between local maxima and minima in the video images are on average λ/2 ≈ 4.5 pixels and that these length scales match with the length scales appearing in the motion artifact patterns. The graph highlights that motion artifacts can quickly emerge even with slight movement over only a few pixels, c.f. Figure 9A. It also highlights the necessity to be able to track movements with sub-pixel precision. Furthermore, the graph highlights that it is important to take spatial length scales into consideration when studying the emergence of motion artifacts. Therefore, we suggest defining a factor, which expresses the strength of the motion in optical mapping videos in comparison to the frequency of contrast or features in the image:

(11) $$\begin{array}{lll} u_{\lambda } & = & \frac{<|\vec{u}|>}{\lambda_{c}} \end{array}$$

Here the frequency of visible features is given as the inverse characteristic length scale λ_*c*_, as computed via the two-dimensional spectral analysis shown in Figure 10C. If *u*_λ_ ≈ 1, then the motion is so large that it will create strong dissociation-related motion artifacts, given that the image contrast *c* is sufficiently high.

Lastly, the quantities *f* and *c* can be extracted from experimental data and used to estimate how likely it is that the video data contains weak or strong motion artifacts. Figures 9D–F show the distributions of the fluorescent signal strengths ρ(Δ*F*), the local contrast ρ(*C*) and the motion strength $\rho \left(\left|{\vec{u}}_{max}\right|\right)$ in the experimental data set shown in Figure 9A. The peaks of the distributions (Δ*F*_*mean*_ = 854 counts, *C*_*mean*_ = 1, 400 counts, $<|{\vec{u}}_{max}|>=3.5$ pixels) were used to determine the signal-to-contrast ratio *f*_*c*_ and the relative motion factor *u*_λ_ for the data set. The likeliness for motion artifacts to occur in this data set is large as *f*_*c*_ < 1 or Δ*F* < *C* and *u*_λ_ ≈ 1 or $<|{\vec{u}}_{max}|>\approx \lambda /2$. Note, that the overall motion in the original recording is moderate ($<|{\vec{u}}_{max}|>\approx 5$ pixels) as it shows the rapidly contracting heart surface during fibrillation. The short distances in the network-like spatial patterns on the surface of the heart can easily generate motion artifacts even when the motion is moderate and in the order of a few pixels (the diameter of the heart being in the order of 100 pixels in our data).

#### 3.2.1. Measuring motion artifact strength based on frequency components in optical mapping video images

As the absolute strength of motion artifacts can not be extracted from experimental data *per se*, we compared motion artifacts appearing in experimental data to synthetic data, which resembled the experimental data as closely as possible, and for which we could compute the absolute values $\left|\vec{u}\right|$, *f*, *c* and λ as described above. Figure 10A shows four normalized synthetic video images depicting a spiral wave pattern (c.f. Figure 3), where the first three images show the electrical pattern being obscured by motion artifacts for different and increasing amplitudes of motion ($<|\vec{u}|>=0.4,0.8,4.8$ pixels) and the last image shows the corresponding tracked and motion-stabilized image with a substantial reduction in motion artifacts (3*rd* image: $<|\vec{u}|>=4.8$ pixels). The synthetic data reproduced the specific signal-to-contrast ratio *f*_*c*_, amplitude of motion $\left|\vec{u}\right|$ or relativ motion *u*_λ_, and texture of the respective experimental data set, see Figure 10C. The lower image sequence shows the spatial frequency contents in the corresponding two-dimensional power spectra. These spectra were computed as averages from all images in each video sequence. The spectra show that with increasing motion and accordingly with increasing motion artifacts, as seen in the upper sequence, the spectral power increases in magnitude for higher frequency components. Comparing both the upper right and lower right images in Figure 10A one finds that the tracking and motion compensation equally reduced motion artifacts and the high-frequency components in the power spectrum. Figure 10B shows the frequency components sampled and averaged along the radial direction within the two-dimensional power spectra (red lines forming a star in sub-image). The graph shows the mean radial profiles P^*^(λ) (averaged from *N* = 8 lines) for different amplitudes of motion ($<|{\vec{u}}_{max}|>=0.1-4.8$ pixels). One can see that the height of the profiles continuously increases with increasing motion strength (light gray: little motion, dark gray or black: up to 5 pixels motion), indicating that higher frequency content becomes larger with increasing motion artifact strengths. For motion above 0.5 pixels each profile exhibits a peak at the characteristic length scale λ, see also Figure 9C. Computing the integral values *r* = ∫P^*^(λ)*dλ* for each profile and plotting the values of *r* over the amplitude of motion $<|{\vec{u}}_{max}|>$ yields the upper curve in Figure 10D. The curve shows a continuous, monotonous increase in *r* that retains a similar shape as the curve in Figure 8B, which indicates that motion artifact strength $\tilde{m}$ depends similarly on the amplitude of motion $\left|\vec{u}\right|$. The graph shown in Figure 10E further emphasizes this dependency, illustrating that the strength of motion artifacts $\tilde{m}$ increases linearly with *r*, suggesting that *r* is a valid measure for the estimation of the magnitude of motion artifacts. This means, that the strength of motion artifacts occurring in experimental data can be estimated by comparing the magnitudes of the frequency content of the spatial motion artifact patterns of synthetic and experimental data with each other. Figure 10D includes two data points (gray dots) computed for the experimental data sets shown in Figure 10C matching the synthetic values (black dots) computed for the same image texture, amount of motion, *f*_*c*_ and *u*_λ_. Comparing the amount of distortion and motion artifacts in the upper left image (motion $<|\overset{\to }{u}|>=0.4$ pixels) and the upper right image (after tracking and stabilization, initial motion $<|\overset{\to }{u}|>=4.8$ pixels) in Figure 9A, one can conclude that the tracking and motion-stabilization yields optical maps, which still include residual motion artifacts comparable in strength to optical maps containing slight motion ($<|\overset{\to }{u}|>=0.4$ pixels). The spectral profiles in Figure 10B confirm this conclusion. The profile obtained for the tracked and motion-stabilized data (green, *r* = 0.4) closely aligns with the profile obtained for very slight motion (light gray, $<|\overset{\to }{u}|>=0.2$ pixels). Also, in Figure 10D all values of *r* for the tracked and motion-stabilized data points (circles) are below *r* < 0.5. This value for *r* is also obtained for data sets with motion smaller than 0.5 pixels (c.f. Figure 10A), lower left image, *r* = 0.45). The data demonstrates that analyzing the spatial frequency content of motion artifact patterns in optical maps, can provide both an estimate for the amount of residual motion artifacts ${\tilde{m}}^{*}$ that are left in motion-stabilized videos after motion tracking, and an evaluation of the accuracy of the tracking, see following section 3.3.

### 3.3. Evaluating motion tracking and motion compensation performance

**Figure 11:**
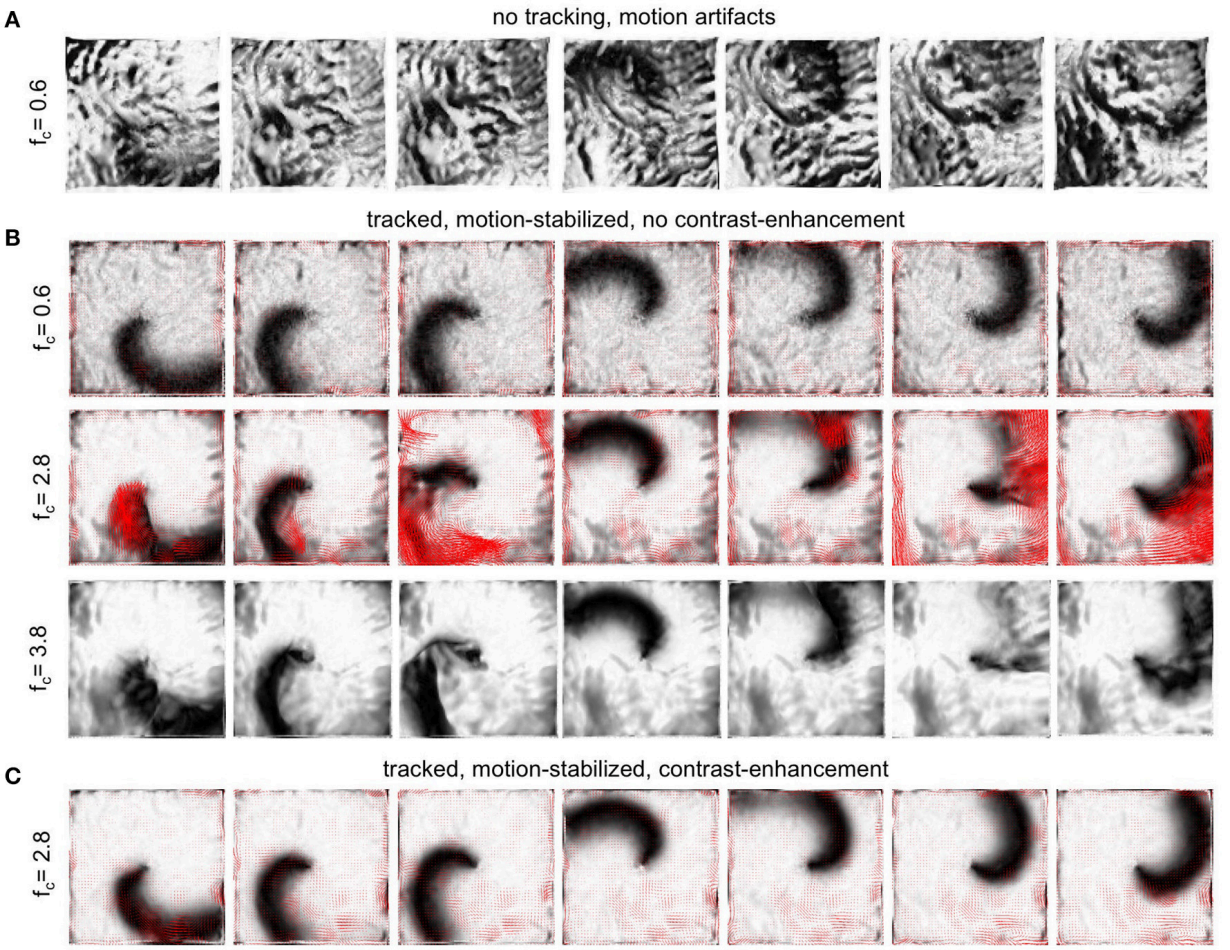
Efficacy of motion compensation and tracking error determined with synthetic optical maps showing contracting, fluorescing cardiac tissue. See also Supplementary Videos 3 and 4. **(A)** Deformed, non-tracked optical maps obscured by motion artifacts for weak to moderate signal strength (*f*_*c*_ = 0.6). **(B)** Motion-stabilized optical maps with substantial artifact reduction for various signal-to-contrast ratios *f*_*c*_ = 0.6, *f*_*c*_ = 2.8, *f*_*c*_ = 3.8 (increasing signal strength) after tracking and warping. Red vectors (row 1 & 2) indicate tracking errors, calculated as mismatches between the tracked and actual simulated tissue configuration. While tracking is sufficiently accurate for small fluorescent signal strengths (*f* < *c*, here *f*_*c*_ < 0.6), the tracking error increases with increasing signal strengths, with mismatches emerging particularly close to the wave front. For very strong signals (*f* > *c*, here *f*_*c*_ = 3.8) the warped, motion-stabilized images become visibly distorted as a result of the erroneous tracking. **(C)** Substantial reduction of tracking errors by introducing contrast-enhancement, amplifying local image gradients to minimize accidental tracking of the electrical wave and the related intensity modulations it causes in the video images. Contrast-enhancement improves the accuracy of the tracking and maintains robust and sufficiently accurate tracking with larger signal strengths (*f*_*c*_ > 2.8), see also Figure 12. All optical maps are pixel-wise normalized over time.

**Figure 12:**
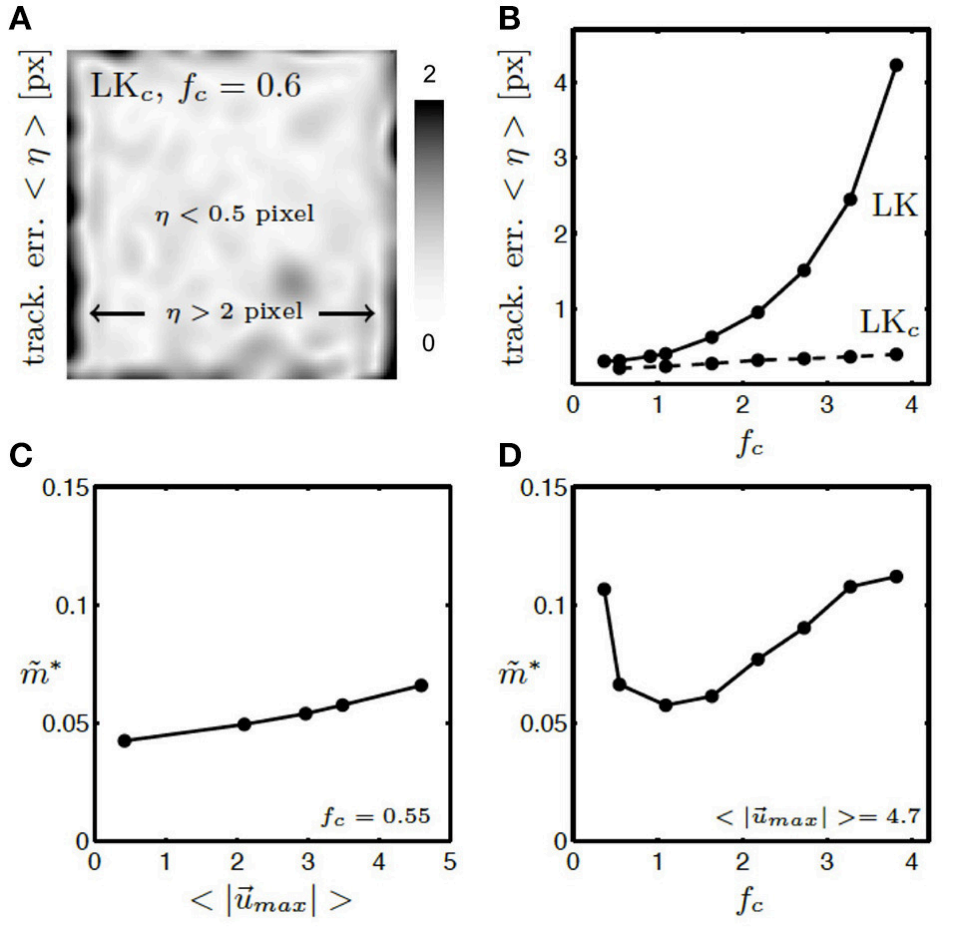
Tracking error η and residual motion artifacts ${\tilde{m}}^{*}$ in synthetic, tracked and motion-stabilized optical maps with various amplitudes of motion and relative signal strengths. **(A)** Tracking error η within video image averaged over all video frames with contrast-enhancement (*LK*_*c*_). Within the video image errors are acceptable (< 0.5 pixel), toward the boundaries errors can become large (η > 2 pixel). Video image is 100 × 100 pixel in size. See also Supplementary Video 4. **(B)** Tracking error η with (*LK*_*c*_) and without (*LK*) contrast-enhancement. Tracking error grows exponentially with increasing signal-to-contrast ratio *f*_*c*_ without image contrast-enhancement (LK) and remains small with image contrast-enhancement (*LK*_*c*_) for all signal-to-contrast ratios *f*_*c*_. **(C)** Residual motion artifacts ${\tilde{m}}^{*}$ are equally small (cf. Figure 10E) for small and large amplitudes of motion in the original videos. **(D)** Residual motion artifacts ${\tilde{m}}^{*}$ are minimal for relative signal strengths in the range of *f*_*c*_ = 0.5 − 2.0 or when *f* ≈ *c*.

To evaluate the performance of the motion tracking and motion compensation algorithm, we assessed how well the algorithm tracks simulated movements of cardiac tissue in the synthetic optical maps, see Figure 11. The efficacy of the motion tracking and motion-stabilization is also demonstrated in Supplementary Video 3. As discussed earlier in this paper, and in the discussion, it is crucial to take into consideration the fluorescent signal when tracking motion in optical mapping videos, as the motion tracking algorithm may confuse the signal with motion-related optical flow and may accidentally track electrical activity instead of motion and deformation. Here we show that with strong fluorescent signal strengths *f* larger than the local image contrast *c* (or large signal-to-contrast ratios *f*_*c*_ > 1), the tracking algorithm may accidentally track the electrical wave phenomena propagating across the heart surface instead of the motion itself. To inhibit such phenomena, we introduced a pre-processing step in our motion tracking scheme, see Figure 4. Using Equation (8), we created locally normalized, contrast-enhanced videos *I*_*c*_(*x, y, t*), in which each pixel is normalized to the maximal and minimal intensity values found within a small disk-shaped sub-region $S_{x,y}$ around the pixel (*x, y*). As a result, the tissue texture or image contrast was maximally intensified, see Figure 4A, and intensity fluctuations caused by the electrical activity were suppressed (see also Figure 4 in Christoph et al., 2017). We tracked both the original video data *I*(*x, y, t*), as well as the contrast-enhanced video data, *I*_*c*_(*x, y, t*) and compared and evaluated the outcome of the tracking in terms of accuracy and robustness. Figure 11 shows how tracking the contrast-enhanced videos outperforms tracking the raw videos and yields good tracking performance for both small and large fluorescent signals. Figure 11A shows the untracked video including heavy motion artifacts (for a signal-to-contrast ratio of *f*_*c*_ = 0.6). In contrast, Figure 11B shows tracked and motion-stabilized optical maps of the same video as shown in Figure 11A, the tracking performed on the raw video *I*(*x, y, t*) without contrast-enhancement. Because of the tracking and motion-stabilization, motion artifacts are substantially reduced and do no longer obscure the electrical wave pattern as seen Figure 11A. Instead, the electrical wave pattern is visible. However, while in the first image sequence with low signal strength (*f*_*c*_ = 0.6) the tracking is accurate, the red arrows indicate the mismatches between the simulated and the tracked tissue configuration, in the two lower image sequences the tracking becomes inaccurate because they contain larger fluorescent signal strengths (2*nd*: *f*_*c*_ = 2.8, 3*rd*: *f*_*c*_ = 3.8). In the bottom most (3*rd*) image sequence the mismatches (red arrows) are not shown. Instead the warped, motion-stabilized images are shown to highlight the distortions that are introduced when warping the original video images using the inaccurate tracking results, see also Supplementary Video 4. In the first image sequence, the tracking was still able to reliably associate tissue regions with each other throughout the image sequence, because the fluorescent signal strength was smaller than the local tissue contrast (*f* < *c*). Mismatches occur only close to the boundaries of the image (cf. Figure 12A). Only mild motion artifacts are recognizable (cf. Figure 8). In the central image sequence (2*nd*) the mismatches (red vectors) between the simulated and tracked tissue configuration are significantly larger than in the first image sequence and occur particularly close to the action potential, suggesting that the algorithm accidentally tracks the electrical wave pattern. The fluorescent signal is significantly larger than the local tissue contrast (*f*_*c*_ = 2.8) and the algorithm is no longer able to associate a tissue region with its own systolic/diastolic or darker/brighter rendition altered through the fluorescence. Even though motion artifacts do not appear to be stronger than in the first image sequence due to the larger relative signal strength, the tracked tissue configuration does not correspond to the real tissue configuration, which consequently makes a mechanical measurement inaccurate. Furthermore, the inaccurate tracking results cause distortions in warped image sequences, as comparably shown in the lower image sequence (*f*_*c*_ = 3.8), when aiming at stabilizing the motion numerically. However, accidental tracking of the electrical wave pattern can be overcome when the tracking is not performed with the original videos *I*(*x, y, t*), but instead with contrast-enhanced videos *I*_*c*_(*x, y, t*), see Figure 11C. For the same video data and signal-to-contrast ratio (*f*_*c*_ = 2.8), as shown in the central image sequence in Figure 11B, the accuracy and robustness of the tracking becomes significantly improved. The mismatches or tracking errors close to the action potential vanish and the mismatches overall are comparably small, just as with small signal-to-contrast ratios, (*f*_*c*_ = 0.6 cf. Figure 11A). All videos in Figure 11 contained the same motion before tracking (initial motion $<|\vec{u}|>=4.7$ pixels).

Contrast-enhanced tracking (LK_*c*_) is accurate and robust and yields small tracking errors for any given signal-to-contrast ratio, see Figure 12B. While without contrast-enhancement (LK) the tracking error η grows exponentially with increasing signal strength or signal-to-contrast ratio, it stays small (η = 0.1 − 0.4 pixels) with contrast enhancement for all signal strengths or signal-to-contrast ratios. Without contrast-enhancement, the tracking does not achieve sub-pixel accuracy (η > 1 pixel) when the fluorescent signal *f* becomes twice as large as the image contrast *c*. The tracking error corresponds to the mismatches between the simulated and the tracked configuration (red vectors, see Figure 11 averaged over all images in the video. Shifts or motion can be tracked with a precision of 0.1 − 0.4 pixels, while the amplitudes of motion were about 10 − 50 times larger in the order of about 5 pixels. We typically observed such amplitudes of motion during arrhythmias and pacing in the experimental data sets (with a sensor size of 128 pixels and the diameter of the ventricle in the order of 100 pixels). As shown in Figure 12A, the tracking error remains small (η < 0.5 pixels) within the entire video image and becomes only larger (η > 2 pixels) close to the boundaries (within ~5 pixels) of the video image. Just as in Figure 11 all tracked and analyzed videos in Figure 12B contained the same motion before tracking (initial motion $<|\vec{u}|>=4.7$ pixels). Nevertheless, the accuracy of the tracking algorithm does not diminish with increasing and stronger motion. Figure 12C shows that the strength of residual motion artifacts ${\tilde{m}}^{*}$ after tracking and motion-stabilization remains constantly small for displacements ranging from 0 − 5 pixels, and the algorithm has demonstrated to reliably detect shifts with larger magnitudes (~10 pixels in video image of size 128 × 128 pixels), see experimental data set shown in Figures 1C,E.

Finally, comparing the experimental and synthetic video data to each other, as shown in Figures 10C–E, it is possible to estimate the accuracy of the tracking in the experiments. Figure 10C shows a substantial reduction in motion artifacts comparing the simulated pre- and post-tracking data. After tracking, the spatial high-frequency content in the optical maps decreased substantially and it is possible to relate this reduction to a reduction in dissociation-related motion artifacts, as shown in Figure 10C, and to consider it as a measure for the precision of the tracking, see also section 3.2.1. In the synthetic data, which matches the experimental data in terms of fluorescent signal and contraction strengths and image contrast, both the amount of motion artifacts $\tilde{m}$ that results with small sub-pixel shifts of the tissue ($|\vec{u}|=0.4$ pixel) and the amount of residual motion artifacts ${\tilde{m}}^{*}$ that is seen after tracking corresponds to the amount of residual motion artifacts ${\tilde{m}}^{*}$ found in the experimental data after tracking. From Figure 10E it is consequently possible to conclude that for the particular data set shown in Figure 10C motion artifacts were reduced by about 75 − 80% ($\Delta \tilde{m}\approx 1-0.04/0.2=0.8$). Furthermore, it can be concluded that the motion tracking algorithm is able to detect shifts with sub-pixel accuracy ($<|\vec{u}|>=0.4$ pixel) in the experimental data set shown in Figure 10C. Figure 12C shows that residual motion artifacts ${\tilde{m}}^{*}$ remain small for both small and large amplitudes of motion, suggesting that similar results could be obtained with other experimental data sets. Figure 12D demonstrates that with varying signal-to-contrast ratios there is an optimum around *f*_*c*_ = 1 for which residual motion artifacts ${\tilde{m}}^{*}$ become minimized after tracking. The graph suggests that for small signal strengths *f* residual motion artifacts become larger simply because with vanishing signal (*f*_*c*_ < < 1) and imperfect tracking it becomes more likely to measure optical flow instead of signal. Likewise, with large signals slight mismatches or tracking errors may lead to an overly strong contribution of the signal to motion artifacts. The regime in which we found the minimum in residual motion artifacts ${\tilde{m}}^{*}$ for signal-to-contrast ratios of *f*_*c*_ = 0.2 − 2.0 is often faced in experimental data sets, for example Di-4-ANEPPS (*f*_*c*_ ≈ 0.5 − 1.0, see for instance Rohde et al., 2005 with *f*_*c*_ ≈ 0.5) or Rhod-2 AM (*f*_*c*_ ≈ 1.0 − 2.0), cf. Figures 9D–F.

## 4. Discussion

In this study, we validated the robustness and accuracy of a 2D marker-free motion tracking and motion stabilization algorithm for performing electromechanical optical mapping studies with beating, fluorescing hearts. Using experimental and synthetically generated optical mapping videos, we compared the tracked data to simulated ground-truth data and found that the algorithm reduces motion artifacts substantially by about 75 − 80% and achieves sub-pixel accuracy (< 0.5 pixels, ~0.2 − 0.4 pixels, see lower curve in Figure 12B) when tracking motion with amplitudes in the range of 1 − 10 pixels (in video images that are in the order of 100 × 100 pixels in size, the heart filling the entire field of view). We further found that the motion tracking algorithm is robust against fluorescence intensity fluctuations which are caused by electrical activity. One of the most important issues in tracking motion in optical mapping videos is the careful disentanglement of motion from fluorescent activity, particularly when using marker-free tracking approaches as in this study. Unlike other tracking algorithms (Seo et al., 2010; Bourgeois et al., 2011; Zhang et al., 2016), the motion tracking algorithm discussed in this paper does not require markers attached to the tissue surface to facilitate the tracking. It instead analyzes and compares anatomical features or landmarks that are visible on the heart surface. With such a marker-free tracking approach, it is important to identify and eliminate factors that could possibly mislead or irritate the tracking and lead to falsely detected shifts or displacements. Such an assessment is particularly important because optical mapping videos, which show fluorescing and contracting cardiac tissue, do not only contain intensity changes that can be attributed to motion (optical flow) alone, but also contain fluorescence intensity fluctuations that are caused by electrical activity, i.e., intensity drops during action potential depolarization or intensity increases during calcium cycling. The motion and fluorescence appear as two superimposed spatio-temporal dark-bright patterns, both of which can be detected by the tracking and therefore need to be disentangled from each other. In the worst case, if the fluorescent signal is large enough, the tracking algorithm may be unable to associate two corresponding image regions between two frames, for example when one video frame shows the tissue during diastole and the other during systole. Even with weak fluorescent signals, such superposition phenomena can lead to tracking errors, which may not be visually evident, see Figures 11, 12. To reconcile this problem, we introduced and applied a contrast-enhancement pre-processing procedure, which intensifies short-scale gradients in video images and suppresses intensity fluctuations caused by electrical activity (Christoph et al., 2017). In this study, we validated that this contrast-enhancement, see Figure 4, creates a unique and robust spatial pattern or texture that can be reliably identified and tracked through video images in the presence of fluorescent signals, just like artificial markers attached to the heart surface. In Figure 11 it is shown that strong fluorescent signals (*f* > *c*) can lead to tracking artifacts, if the tracking is performed without contrast-enhancement. The tracking artifacts may arise when the original, unprocessed video image is tracked and the algorithm uses the local grayscale pattern to uniquely identify a particular tissue segment and follow its motion through the image plane. However, this original spatial intensity pattern is superimposed or modulated by fluorescence intensity changes that typically occur when the tissue is loaded and imaged, for instance, with voltage- or calcium-sensitive dyes. In Figures 11, 12B, we show that if these modulations become large, they can alter the image in a way that the algorithm — without further precautions—is unable to match the local spatial intensity pattern associated with one tissue region with its corresponding deformed spatial pattern in a different video frame. As a result, the algorithm produces tracking errors with increasing fluorescent signal strengths (Δ*F*/*F*). In the worst case, with very strong fluorescent signals, the tracking algorithm could accidentally track the movements of action potential or calcium waves across the surface instead of motion, as demonstrated in Figure 11B. Using synthetic data, we verified that with contrast-enhancement the tracking achieves sub-pixel precision for arbitrary fluorescent signal strengths *f*, see Figure 12A and lower curve in Figure 12B. Tracking the contrast-enhanced videos, we were able to image strongly beating and contracting hearts stained with voltage-sensitive dye (Di-4-ANEPPS) and obtain co-moving optical maps showing action potential waves propagating across the heart surface with substantially inhibited motion artifacts. At the same time, we were able to ensure that we performed an accurate measurement of the time-varying mechanical configuration χ(*t*) of the tissue surface visible within the video images as we verified that tracking errors remain low, see Figure 12B.

The main advantage of our method is that we can image the beating heart without having to attach markers to its surface. At the same time, the algorithm is fully automatic and does not require any manual supervision, i.e., manual selection of markers or image features to initiate or enable the tracking is not necessary. Motion tracking (Matlab), warping and resampling and other processing (custom C++ code) requires approximately 1 min of computation time per video image on a single CPU. We do, however, anticipate that the tracking and motion-stabilization could also be performed much faster (in the order of seconds or even milliseconds per video frame) using parallel computing and streamlining the procedures. In this study, we analyzed relatively small video images with sizes of 100 × 100 or 128 × 128 pixels. Videos with such sizes are produced by state-of-the-art cameras (MiCAM ULTIMA camera, SciMedia, Japan: 100 × 100 pixels; Evolve 128 camera, Photometrics Inc., USA: 128 × 128 pixels). The tracking can also be performed with video data recorded with cameras with much larger sensors, given that the video properties (noise level, image gradients, density or length scales of image features) are comparable and do simply scale with the size of the video image. Note thus that the different amplitudes of motion, which we observed during fibrillation (approximately 1 − 5 pixels), tachycardia or pacing (approximately 5 − 15 pixels), and sinus rhythm (approximately 10 − 30 pixels) would scale with the image sensor size if the heart filled the field of view and could be stated in calibrated units (*mm*). The tracking is also generally applicable to other data obtained with different setups, species (we successfully applied the algorithm to data obtained with rabbit, pig, mouse and alligator hearts) or dyes (we used Di-4-ANEPPS, Di-4-ANBDQPQ, Rhod2-AM, Fluo-3). Due to the single-camera imaging setup, we were only able to image planar movements within the video images, see Figures 1A,B. However, using the same tracking algorithm and a multi-camera setup, we previously demonstrated that the three-dimensional motion and deformation of the heart surface can also be captured and that action potential waves can be mapped on large (180°) and strongly curved parts of the deforming ventricular walls (Christoph et al., 2017). As in the present study, the 2D motion tracking algorithm was used to detect two-dimensional displacements in the video images, and afterwards the 2D data was used to compute three-dimensional displacements combining the data from multiple cameras. While the aim in the multi-camera study was to provide a proof-of-concept that three-dimensional electromechanical optical mapping is possible, our aim in the present study is to discuss the performance of the two-dimensional tracking itself. We verified, with the aid of synthetic video data, that the tracking of the tissue's mechanical configuration is accurate and robust. The robustness is demonstrated in the tracking's ability to produce displacement vector fields, which describes a smooth and continuous movement of the tissue through space, even though each video frame was registered individually and independently. This has implications for both 2D and 3D imaging alike, as the 2D tracking data is the basis for the 3D reconstruction discussed in Christoph et al. (2017).

Motion artifacts can be reduced substantially using numerical motion tracking and motion compensation techniques, as shown in this and in previous studies (Christoph, 2015; Zhang et al., 2016; Christoph et al., 2017, 2018). However, judging from the outcome of the motion tracking and motion compensation alone, it is not immediately apparent how accurate the motion was tracked. Furthermore, it is difficult to quantify the amount of residual motion that may still be present in motion-stabilized videos or to determine to what extent motion artifacts were reduced. Motion artifacts are well known to manifest as distortions or deflections in optical traces (Rohde et al., 2005; Christoph, 2015; Christoph et al., 2017) and become immediately apparent particularly during sinus rhythm, as during sinus rhythm motion alters the very characteristic shape of the action potential. A quantitative assessment of motion artifacts and potential deviations of the optical traces from the true action potential remains difficult, as ground-truth data is unknown. In particular during arrhythmias, the identification of motion artifacts is not trivial because action potentials can take on various and less specific shapes than during sinus rhythm. To better understand the origins of motion artifacts, we generated synthetic motion artifact patterns and studied their properties and dependence on motion and other features of the video data. We found that a spectral analysis of motion artifact patterns in optical maps can be used to estimate the residual error of the tracking and the amount of residual motion and motion artifacts. We also found that the strength of dissociation-related motion artifacts are mainly determined by the ratio of the fluorescent signal strength *f* in comparison to the local image contrast *c* and the ratio of the amplitude of motion $\left|\vec{u}\right|$ in comparison to the length scales λ of image features. In determining these video properties alongside a spectral analysis of motion artifacts, one can evaluate the efficacy of motion-stabilization and motion artifact compensation algorithms. In the synthetic data, the amount of residual motion artifacts is a direct measure for the accuracy of the tracking, see Figure 10E. Using a simplistic computer model, we were able to create optical maps, which reproduced the most essential aspects of an optical mapping video. In future work, it may be necessary to simulate the full three-dimensional heart together with a three-dimensional imaging scene and the positioning of different light sources within that scene. Taking into account a more realistic imaging situation is necessary in order to simulate the generation of illumination-related motion artifacts caused by movements of the heart inside an inhomogeneously illuminated scene. Inhomogeneous illumination or, more precisely, relative motion between the heart and light sources can cause illumination-related motion artifacts, which add to dissociation-related motion artifacts. Illumination-related motion artifacts can not be overcome by tracking, but can be compensated by ratiometric imaging (Brandes et al., 1992; Knisley et al., 2000; Hooks et al., 2001; Tai et al., 2004; Bachtel et al., 2011; Bourgeois et al., 2011; Zhang et al., 2016) or numerical light-field correction techniques (introduced in Christoph et al., 2017). Here, we neglected illumination-related motion artifacts, because in the experiments we typically illuminated the hearts “flat”, meaning that we avoided larger intensity gradients across the images and tried to illuminate the heart surface as evenly as possible with multiple LEDs from all sides. We experienced that with flat illumination and small amplitudes of motion (1 − 10 pixels) illumination changes do not pose greater issues. Nevertheless, in future work, illumination-related motion artifacts will have to be considered more carefully.

Performing optical mapping experiments with beating hearts requires careful handling of the tissue preparations. For instance, it is very important, and much more so than during conventional optical mapping without motion, to avoid dust or Tyrode stains on the glass walls through which the imaging is performed. The avoidance of particles or bubbles flowing inside the bath is also necessary. Both dust or stains on the glass walls and particles and bubbles moving through the field of view may accidentally be tracked or may compromise tracking. Imaging the heart from the top through the surface of the Tyrode solution may be prohibited by ripples that form on the water surface when the heart contracts. The tracking may also pick up the flickering of instable light sources. Strongly contracting tissue preparations may require mechanical fixation to inhibit excessive motion. Especially during sinus rhythm, the heart may rotate or move out of the field of view such that its motion can not be captured with a single camera. At the same time, one needs to be very careful when trying to fix it in one location. Subjecting the heart wall to mechanical pressure or bringing it in mechanical contact with instrumentation could lead to blockage of its vascular system and improper perfusion and ischemia. We experimented with molds and flexible holders to mechanically restrict the hearts, but, due to repeated complications with proper perfusion, have resorted to freely moving hearts, which are simply attached to the perfusion outflow.

## 5. Conclusions

We demonstrated that optical mapping can be performed with strongly contracting isolated hearts using computer vision techniques. Without using artificial markers attached to the heart surface, we tracked and numerically stabilized the motion of the beating heart to measure electrophysiological wave phenomena propagating across the contracting heart surface in a co-moving frame of reference. We validated the robustness and accuracy of the marker-free motion tracking and motion compensation algorithm using synthetically generated optical mapping videos and found that the algorithm achieves sub-pixel accuracy, reduces motion artifacts substantially and is unaffected by intensity modulations in the video images caused by electrical activity. As a result, it becomes possible to perform electromechanical optical mapping with beating hearts without having to attach markers to the heart. Furthermore, we found that (residual) motion artifacts can be used as a direct measure for the accuracy of the tracking.

## Data availability statement

The datasets generated and analyzed for this study are available from the corresponding author upon reasonable request.

## Author contributions

JC and SL designed the research. JC designed, implemented and conducted the simulations and analyzed all numerical data. JC conducted the experiments and analyzed all data. JC wrote and revised the manuscript. Both authors read and approved the submitted version.

### Conflict of interest statement

The authors declare that the research was conducted in the absence of any commercial or financial relationships that could be construed as a potential conflict of interest.

## Supplementary material

Supplementary Video 1 Tracking of contracting and deforming left ventricular heart surface of a rabbit heart during pacing and the onset of ventricular fibrillation. Points (black) indicating in-plane displacements of single tissue segment with respect to reference position (gray).

Supplementary Video 2 Action potential wave propagating across contracting and deforming left ventricular surface of a rabbit heart.

Supplementary Video 3 Synthetic optical maps showing action potential spiral wave (Δ*F/F* > 10%, exaggerated) on contracting tissue before and after tracking and motion-stabilization.

Supplementary Video 4 Tracking errors in synthetic optical maps without and with contrast-enhancement for a signal-to-contrast ratio of *fc* = 2.8, c.f. Figure 11. Without contrast-enhancement the tracking and motion-stabilized videos contain errors and heavy distortions respectively. Contrast-enhancement reduces mismatches between real simulated and tracked tissue configurations (red vectors) significantly.
